# Sustainable Microwave‐Assisted Synthesis of Medium‐ and Long‐Chain Alkyl Levulinates from Biomass‐Derived Levulinic Acid

**DOI:** 10.1002/cssc.202402508

**Published:** 2025-03-17

**Authors:** Alberto J. Huertas‐Alonso, Diego J. González‐Serrano, Manuel Salgado‐Ramos, Milad Hadidi, Prado Sánchez‐Verdú, Beatriz Cabañas, Christopher J. Chuck, James H. Clark, Andrés Moreno

**Affiliations:** ^1^ Departamento de Química Inorgánica Orgánica y Bioquímica Facultad de Ciencias y Tecnologías Químicas Universidad de Castilla-La Mancha Avenida Camilo José Cela n°10 13005 Ciudad Real Spain; ^2^ Departamento de Química Física, Facultad de Ciencias y Tecnologías Químicas Universidad de Castilla-La Mancha Avenida Camilo José Cela s/n 13071 Ciudad Real Spain; ^3^ Present address: Department of Materials and Environmental Chemistry Stockholm University SE-106 91 Stockholm Sweden; ^4^ Instituto de Combustión y Contaminación Atmosférica (ICCA) Universidad de Castilla-La Mancha Universidad de Castilla-La Mancha Camino de Moledores s/n 13005 Ciudad Real Spain; ^5^ Department of Chemical Engineering University of Bath Bath BA2 7AY United Kingdom; ^6^ Department of Chemistry Green Chemistry Centre of Excellence University of York Heslington, York YO10 5DD UK; ^7^ Present address: Research group in Innovative Technologies for Sustainable Food (ALISOST), Department of Preventive Medicine and Public Health, Food Science, Toxicology and Forensic Medicine, Faculty of Pharmacy and Food Sciences Universitat de València Avenida Vicent Andrés Estellés s/n Burjassot, València 46100 Spain

**Keywords:** Alkyl levulinates, Biofuel, Green chemistry, Microwave chemistry, Renewable resources

## Abstract

Alkyl levulinates (ALs) represent a family of bio‐compounds derived from levulinic acid (LA), a platform chemical obtained from lignocellulosic biomass. Medium‐ and long‐chain ALs (pentyl levulinate or longer) have shown potential as biofuel and fuel additives due to their relatively low oxygen content and resemblance to biodiesel. This study reports a fast and environmentally friendly method for synthesizing ALs via microwave (MW)‐assisted LA esterification, laying emphasis on medium‐ and long‐chain ALs. By combining *p*‐toluenesulfonic acid (5 wt % loading) as catalyst and MW radiation as heating source for a short time (5 minutes), excellent yields of ALs (≥89 mol %) were achieved for a wide range of primary and secondary alcohols (2–10 carbons), overcoming the expected lower reactivity of long chain alcohols. Additionally, formation of undesired side products, such as dialkyl ethers or LA aldol condensation products, was significantly minimized. The feasibility of recovering the unreacted alcohol was successfully proved by simple distillation (88 wt % recovery). The green chemistry metrics assessment proved that this approach aligns with the green chemistry principles and the United Nations Sustainable Development Goals, offering a more sustainable pathway for biofuel and fuel additive production.

## Graphical abstract

Green chemistry assessment of an environmentally friendly microwave‐assisted (MW) esterification of levulinic acid to obtain medium‐ and long‐chain alkyl levulinates. Synthesis of biofuels and fuel additives from renewable sources in high yields (>90 mol %).

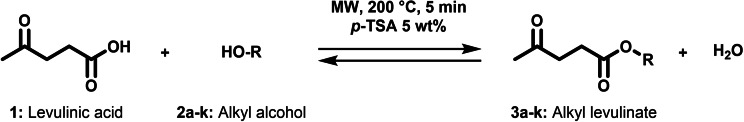

## Introduction

Throughout history, most of the global energy production and chemical manufacturing has heavily relied on non‐renewable fossil‐carbon sources, since they were abundant and relatively cheap.^[1]^ However, in recent years, the escalating demand for fuels, materials and chemicals has led to a significant increase in the dependence on fossil‐carbon reserves. Crude oil perfectly exemplifies this trend, with a consumption around 101 million barrels per day in 2023.^[2]^ Consequently, the overexploitation of these natural resources and the surge of greenhouse gas emissions derived from their combustion are posing a global threat to the environment. Therefore, there is an urgent need for a paradigm shift to tackle these issues. In this regard, lignocellulosic biomass has emerged as a sustainable alternative feedstock for energy and chemical production.^[3]^ Lignocellulose, the most abundant type of biomass, primarily consists of cellulose, hemicellulose, and lignin. In addition, lignocellulosic biomass possesses a net‐zero carbon footprint, balancing CO_2_ emissions through its fixation.^[4]^

Alkyl levulinates (ALs) comprise a group of valuable bio‐compounds derived from lignocellulosic biomass, concretely from levulinic acid (LA). LA is obtained through the depolymerization and dehydration of carbohydrates in acidic media (e. g. cellulose).[^5^, ^6^] LA has exhibited enormous potential as precursor of a wide range of chemicals,^[7]^ being listed among the top‐12 biomass‐derived platform chemicals.^[8]^ Nowadays, ALs are attracting great interest as a sustainable substitute for fossil‐carbon derived products due to their versatile applications.^[9]^ The most studied application for ALs is as fuel additives. For instance, methyl and ethyl levulinates showed good performance as anti‐knock additives,^[10]^ enhancers of lubricity and conductivity in diesel fuel,^[11]^ or reducing the post‐combustion fumes.^[12]^ Nevertheless, their low cetane number and limited miscibility with diesel fuels at low temperatures pose a drawback to their extensive use. Miscibility issues could be mitigated by using long‐chain ALs, since their structure resembles to that of biodiesel, and their relatively low oxygen content enhances energy density and hydrophobicity.[^11^, ^13^, ^14^] Other reported applications of ALs include environmentally friendly solvents,[^15^, ^16^, ^17^] polymer synthesis^[18]^ or fragrances and flavorings.^[19]^

Regarding the synthesis of ALs, esterification of LA is the most straightforward approach.^[9]^ Historically, mineral acids, such as HCl or H_2_SO_4_, were commonly used as catalyst in esterification reactions. However, the trend nowadays is to replace corrosive mineral acids with more sustainable alternatives. In this line, heteropolyacids (HPAs)[^20^, ^21^, ^22^, ^23^, ^24^, ^25^, ^26^, ^27^] and zeolites[^28^, ^29^, ^30^] have received significant attention. However, HPAs normally need to be supported on zirconia,[^21^, ^22^] graphitic nitrides,^[23]^ molecular sieve,^[24]^ zeolites[^25^, ^26^] or clays^[27]^ to achieve high LA conversion, while classic zeolites have exhibited limited catalytic activity in ALs synthesis.^[9]^ Nevertheless, mesoporous zeolites[^28^, ^29^] or desilication processes^[30]^ have shown potential for enhancing their catalytic activity. In this regard, *p*‐toluenesulfonic acid (*p*‐TSA) could help to overcome all these issues. Its p*K_a_* value in water is similar to that of H_2_SO_4_, and important feature for esterification reactions, and its solid form makes it easier and safer to handle. Moreover, *p*‐TSA has gained attention as a catalyst for lignocellulosic biomass valorization, since its full recovery has been successfully proven.[^31^, ^32^, ^33^] Therefore, the development of *p*‐TSA‐catalyzed processes would be valuable for making bio‐based products.

In addition to developing more sustainable catalytic procedures, the search of high‐efficiency heating systems also plays a key role. In this regard, microwave (MW) radiation has emerged as a prominent non‐conventional heating system in the realm of organic synthesis.[^34^, ^35^] The volumetric heating mechanism of MW radiation results in a more homogenous and faster heating of the sample, decreasing notably reaction times and undesired side‐reactions.^[36]^ However, since MW has proven its potential at laboratory scale, there are still challenges related to its scale‐up,^[37]^ mainly related to the penetration depth of MW in large volumes.^[38]^ To overcome this drawback and implement MW at industrial scale, efforts are heading towards the combination of MW with continuous flow reactors.[^39^, ^40^]

Despite the aforementioned promising properties of medium‐ and long‐chain ALs for fuel applications, there are still challenges to address regarding their synthesis to align it with the green chemistry principles and Sustainable Development Goals (SDGs) (Table 1). Some of the identified challenges are the long reaction times needed (≥8 h),[^24^, ^25^, ^41^, ^42^, ^43^, ^44^] what involves high energy consumption; excessive chemicals usage (high catalyst loading or high alcohol ratios);[^13^, ^24^, ^28^, ^29^, ^42^, ^45^] or the use of hazardous chemicals.^[13]^ Even though MW radiation could overcome some of the aforementioned issues, its use as heating source for the synthesis of medium‐ and long‐chain ALs has received little attention, and the examples available in the literature still required long reaction times[^46^, ^47^] or high alcohol:LA ratios and catalyst loadings.[^46^, ^48^]

**Table 1:** Literature review about medium‐ and long‐chain (pentyl levulinate or longer) alkyl levulinates synthesis via levulinic acid esterification. LA: levulinic acid. AL: alkyl levulinate. MW: microwave heating.

Entry	Molar ratio LA:alcohol	Catalyst (loading)	Temperature (°C)	Reaction time	Alcohol	LA conversion, *AL yield*	Ref.
1	1 : 5	WCl_6_ (10 % mol; 34 wt %)	70	15 min	1‐hexanol	100 %, *88 %*	^[13]^
			100	75 min	2‐hexanol	100 %, *82 %*	
			100	75 min	3‐hexanol	99 %, *88 %*	
			80	60 min	2‐ethyl‐1‐butanol	100 %, *88 %*	
			100	60 min	Cyclohexanol	100 %, *91 %*	
			100	30 min	1‐octanol	n.d., *82 %*	
2	1 : 10	Supported silicotungstic acid (25 wt %)	70	10 h	1‐pentanol	85 %, *n.d*.	^[24]^
					1‐hexanol	100 %, *n.d*.	
3	1 : 2	12‐Tungstophosphoric acid anchored to MCM‐22 (1.86 wt %)	90	8 h	1‐pentanol	70 %, *67 %*	^[25]^
					1‐hexanol	77 %, *74 %*	
					1‐octanol	78 %, *76 %*	
4	1 : 7.56	Zeolite H‐ZSM‐5 (25.4 wt %)	120	4 h	1‐octanol	n.d., *99 %*	^[28]^
5	1 : 9	Zeolite H‐ZSM‐5 (24 wt %)	128	5 h	1‐hexanol	n.d., *97 %*	^[29]^
6	1 : 3	Sulfonic acid (0.17 g)	140	8 h	1‐hexanol	100 %, *84 %*	^[41]^
7	1 : 10	Sulfonic acid (10 mol %; 70 wt %)	90	24 h	1‐pentanol	92 %, *88 %*	^[42]^
					1‐octanol	84 %, *n.d*.	
8	n. d.	organosilica nanoflowers (n.d.)	50	24 h	1‐octanol	n.d., *91 %*	^[43]^
					1‐dodecanol	n.d., *96 %*	
9	1 : 1	Zr‐MOF (9 mol % Zr)	80	20 h	1‐dodecanol	n.d., *90 %*	^[44]^
					1‐hexadecanol	n.d., *82 %*	
					Oleyl alcohol	n.d., *62 %*	
10	1 : 3	Sulfated silica nanocatalyst (69 wt %)	90	7 min	1‐hexanol	95 %, *93 %*	^[45]^
11	1 : 5	Preyssler catalyst (86 wt %)	120 (MW)	3 h	1‐pentanol	67–74 %, *n.d*.	^[46]^
					1‐hexanol		
					1‐octanol		
					1‐decanol		
12	1 : 2	molten salt hydrate AlCl_3_+LiCl ⋅ 3H_2_O (15 mL LiCl ⋅ 3H_2_O+34 wt % AlCl_3_)	150 (MW)	3 h	1‐hexanol	n.d., *95 %*	^[47]^
					1‐octanol	n.d., *93 %*	
13	1 : 20	Sulfonic acid (5 wt %)	120 (MW)	30 min	1‐pentanol	n.d., *95 %*	^[48]^
14	1 : 6	*p*‐toluenesulfonic acid (5 wt %)	200 (MW)	5 min	11 examples (C2‐C10)	89 – 99 %, *89 – 99 mol %*	This work

n.d.: not disclosed

Given the background, it is envisaged a need for further research to develop short and energy‐efficient catalytic procedures to obtain medium‐ and long‐chain ALs. To fill that void, herein is presented a MW‐assisted esterification of LA, with emphasis on medium‐ and long‐chain ALs, since their resemblance to biodiesel make them perfect candidates for fuel and energy applications. In the pursue of that goal, the influence of several reaction parameters (time, LA:alcohol molar ratio, type of a catalyst and its loading) on MW‐assisted esterification of LA with hexanol were carefully assessed. In addition, special attention has been paid to side reactions that could lead to tedious separation processes, minimizing the sustainability impact of the method.

Finally, the assessment of green chemistry performance metrics (GCPMs) demonstrated that this work would pave the way for a more cost‐effective synthesis of relevant bio‐based compounds with application as fuel additives and biodiesel. This is aligned with SDGs, not only with SDG 7, which aims for affordable and clean energy, but also SDG 12 (sustainable consumption and production patterns of chemicals and waste) and SDG 13 (climate action with the obtention of fuels from renewable sources).

## Results and Discussion

**Figure 1:**
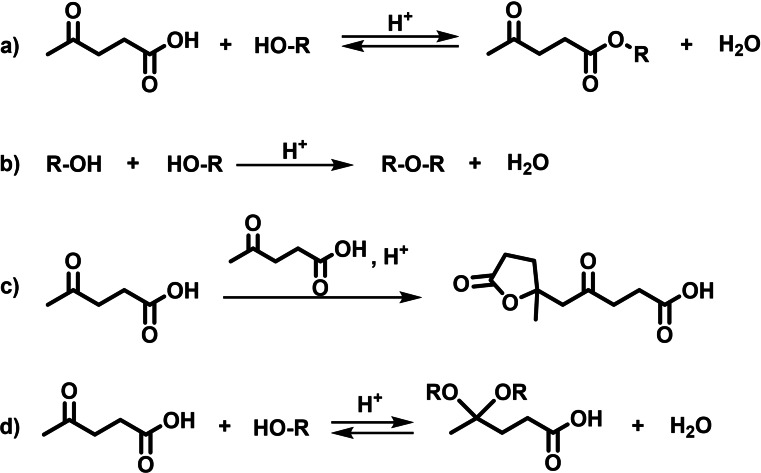
General reaction scheme for levulinic acid esterification a), autocondensation reaction of alcohol b), aldol condensation of levulinic acid c) and acetal/hemiacetal formation d).

The aim of this study was to maximize the ALs yield obtained through MW‐assisted esterification of LA (Figure 1a), laying emphasis on medium‐ and long‐chain ALs due to their promising properties for applications in biofuel and fuel additives. Additionally, special attention should be paid to possible side reactions, since in acidic media the alcohol could undergo autocondensation to yield the corresponding dialkyl ether (Figure 1b), while LA could undergo aldol condensation (Figure 1c) and/or acetal formation in the ketone (Figure 1d). These side reactions pose a detrimental impact on the yield and purity of AL, and must be minimized in a great extent to circumvent tedious separation processes, thus ensuring the feasibility of the method at industrial scale.

## Side Reactions Assessment

As depicted in Figure 1, there are three side reactions in the acid‐catalyzed esterification of LA that should be considered. Firstly, the etherification reaction of alcohol (Figure 1b) generates the corresponding dialkyl ether. This reaction forms water, which can potentially hydrolyze the ester, shifting the equilibrium back towards the reactants (Figure 1a). Consequently, this could negatively affect the formation of the desired product. This side reaction during LA esterification has received limited attention in the literature, with only a few authors reporting on the extent of dialkyl ether formation.^[49]^ For instance, the conversion of ethanol into diethyl ether is promoted by high temperatures and catalyst loadings, as well as long reaction times.^[50]^ Also, high conversions of methanol (28 %) and ethanol (16 %) into dimethyl and diethyl ether, respectively, were reported using silicotungstic acid as catalyst.^[51]^ In contrast, it is worth mentioning the low alcohol conversion to dialkyl ether (2 %) reported by Peng *et al*., when using sulfated titania materials^[52]^ or low sulfuric acid concentrations.^[53]^ Hence, minimizing the formation of dialkyl ethers during LA esterification is crucial, as it not only reduces AL yield, but could also lead to challenging separation processes and issues during recycling of unreacted alcohol.^[9]^ However, recent studies suggested that despite these drawbacks, the high cetane number of dialkyl ethers makes them suitable for diesel additive applications. Thus, small quantities of dialkyl ethers are considered acceptable in AL additives.[^14^, ^53^, ^54^]

Secondly, the aldol condensation of LA (Figure 1c) should be also carefully assessed. The main aldol condensation product of LA is depicted in Figure 1c, however, this reaction could follow different pathways, as shown in Scheme S2.[^55^, ^56^] Monitoring this side reaction is essential because, despite achieving high LA conversions, the yield of AL may remain low. Therefore, to inspect whether aldol condensation of LA occurred in the method used in this study, an initial assessment of the possible products from this reaction was conducted.

To this end, LA was subjected to MW radiation at 200 °C for 5 minutes in presence of *p*‐TSA and without any alcohol. ^1^H NMR spectroscopy (Figure S3) corroborated the formation of product 4 (Scheme S2), which is the main aldol condensation product expected, as described by Amaransekara *et al*.^[55]^ This information allowed us to identify possible side‐products signals in the NMR spectrum of every reaction carried out during this study. Comparison of NMR spectra revealed that no aldol condensation products were formed in this procedure, even under the reaction conditions more favorable to this aldol reaction, such as high LA concentration (Figure S4) or long reaction time (Figure S5).

Finally, the formation of acetal and/or hemiacetal has been also considered. This side reaction was expected to be less relevant, since it mainly occurs with 1,2‐ or 1,3‐diols due to the stability of the 5‐ or 6‐membered ring final product.[^57^, ^58^, ^59^, ^60^, ^61^, ^62^] However, in the literature can be found examples of dimethylketals.^[63]^

To assess the possible formation of acetals, 2‐butanone was selected as an alternative to LA (to avoid the interference of the esterification reaction) due to their resemblance, and subjected to the reaction conditions employed in this study in presence of hexanol. ^1^H NMR spectra (Figure S6) revealed that there was no formation of acetal/hemiacetal. After reaction, there are not new signals from 2‐butanone acetals, as would be expected if the acetal was formed, since 1‐H and 3‐H protons would be shifted to a higher field.^[58]^

Thus, since the only LA‐derived product obtained in this procedure is the corresponding levulinate ester, it could be assumed that the LA conversion would be equivalent to the AL yield.

## Optimization

**Reaction time**. Firstly, the influence of reaction time on hexyl levulinate and dihexyl ether formation was assessed. The initial reaction parameters were set as follows: 200 °C as reaction temperature, 5 wt % *p*‐TSA as catalyst, and LA:hexanol molar ratio 1 : 6. Because of medium‐ and long chain ALs entail the main interest of this work, 200 °C was selected as reaction temperature and kept constant since is a value above or near to the reflux temperature of the corresponding alcohols. Several studies highlighted the benefit of reaction temperatures above or near to the reflux temperature of the alcohol in the MW‐esterification of LA, attributing this to the endothermic nature of the esterification reaction,[^30^, ^48^] and suggesting that higher temperatures could promote LA conversion in shorter reaction times.[^20^, ^64^]

**Figure 2:**
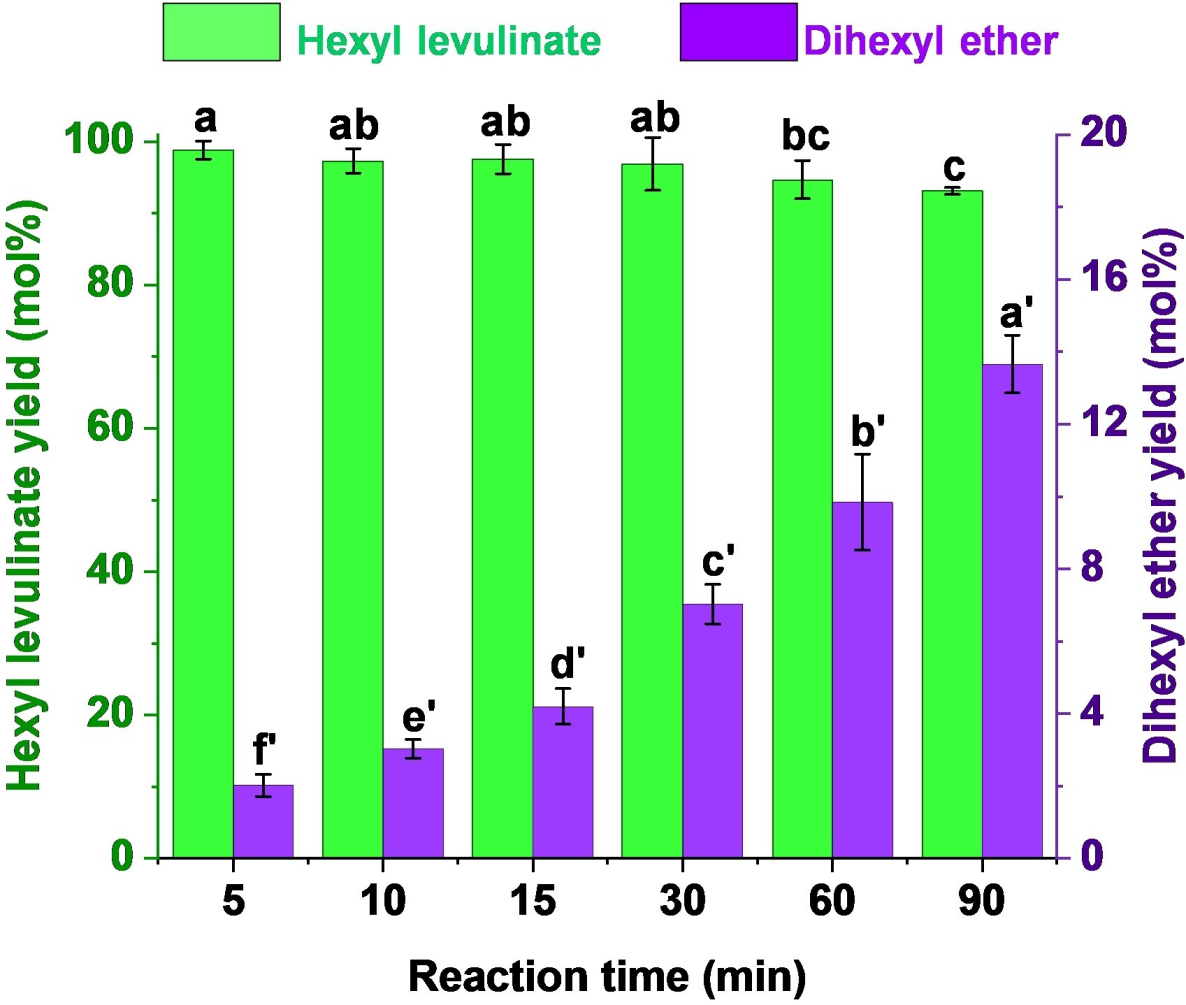
Dependence of hexyl levulinate and dihexyl ether yields on reaction time. Reaction conditions: 200 °C, *p*‐toluenesulfonic acid (*p*‐TSA, 5 wt %) as catalyst, levulinic acid (LA):hexanol molar ratio 1 : 6. *Results are represented as means ± SD, n=3 and values within each variable with the different letters are significantly different (p<0.05).

The use of MW radiation as heating source allows shorter reaction times. As illustrated in Figure 2, an excellent 98 mol % yield of hexyl levulinate was achieved in only 5 minutes of reaction time, in contrast with other studies that required 3 hours to obtain high hexyl levulinate yield (95 %).^[47]^ The decrease in hexyl levulinate yield at long reaction times (93 mol % at 90 minutes) was likely attributed to the higher extent of dihexyl ether formation (14 mol % yield at 90 minutes). The ether formation also generates water (Figure 1b), and according to Le Chatellier′s principle, promotes the ester hydrolysis (Figure 1a). Additionally, dihexyl ether yield exhibited a direct clear time‐dependence, being higher at long reaction times. This higher formation of dihexyl ether at long reaction times was expected, and is consistent with the observations made by other authors.[^49^, ^50^]

Summarizing, significant differences in hexyl levulinate yield from 5 to 30 minutes reaction time were not observed. However, the extent of dihexyl ether formation significantly increased at longer reaction times (Figure 2). For that reason, 5 minutes was selected as the optimal reaction time for further parameter studies, since afforded the highest hexyl levulinate yield (98 mol %) while keeping a low dihexyl ether yield (~2 mol %), a value that would be considered acceptable for future implementation of the process at industrial, as stated by other authors.^[53]^

**Figure 3:**
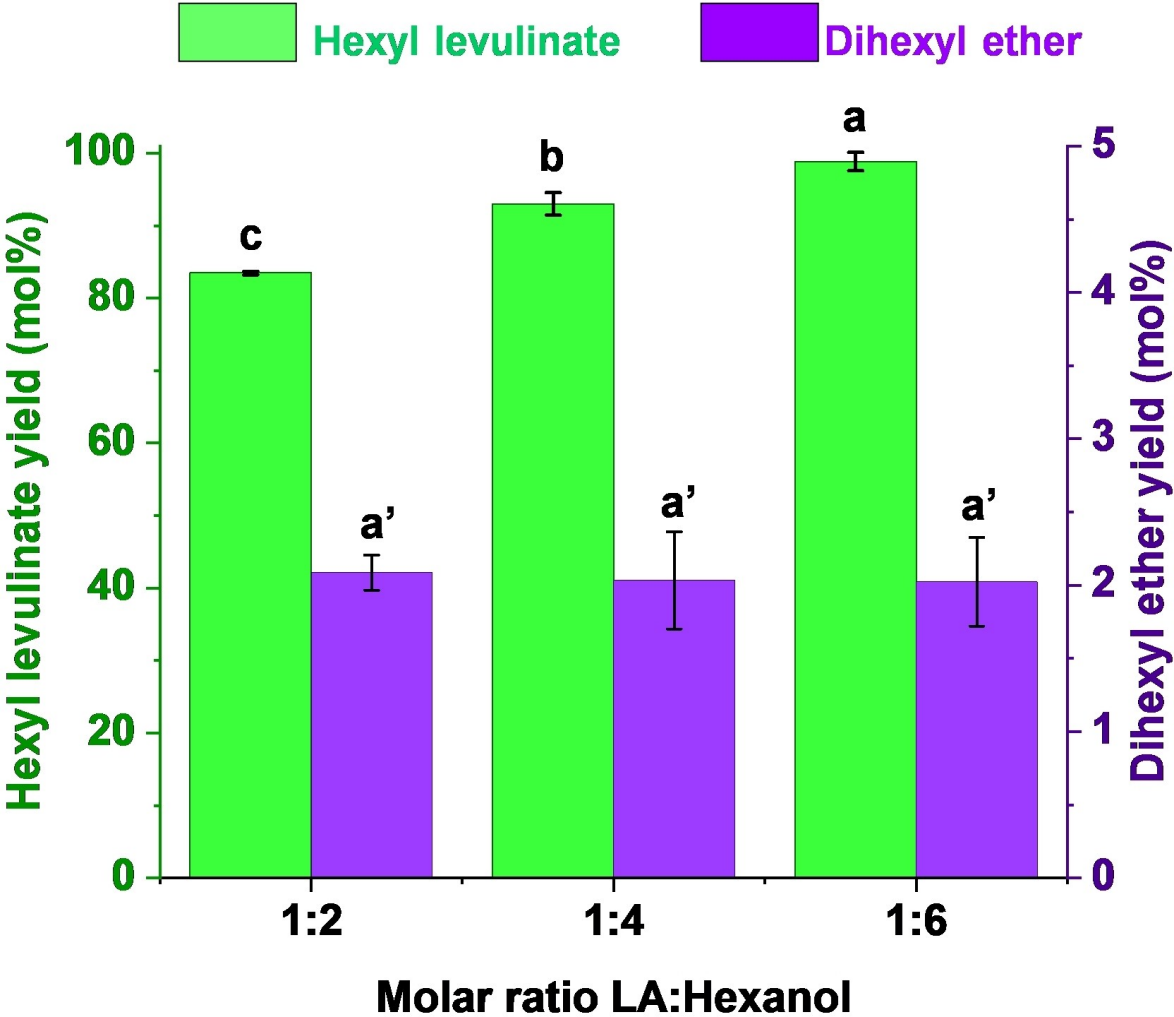
Dependence of hexyl levulinate and dihexyl ether yields on levulinic acid (LA):hexanol molar ratio. Reaction conditions: 200 °C, 5 minutes reaction time, *p*‐toluenesulfonic acid (*p*‐TSA, 5 wt %) as catalyst. *Results are represented as means ± SD, n=3 and values within each variable with the different letters are significantly different (p<0.05).

**LA/alcohol molar ratio**. As depicted in Figure 1a, esterification reactions are chemical equilibriums governed by Le Chatelier′s principle. Therefore, to enhance the yield of the ester product, either one of the reactants should be used in large excess or water should be removed from the reaction media as it forms. To maximize the hexyl levulinate yield while using the lowest excess of hexanol possible, different molar ratios LA:hexanol were tested from 1 : 2 to 1 : 6. As observed in Figure 3, there is a clear correlation between hexyl levulinate yield and the molar ratio between LA and hexanol. The LA:hexanol molar ratio 1 : 6 afforded the highest hexyl levulinate yield (98 mol %). In addition, the formation of dihexyl ether seems to be independent on the LA:hexanol molar ratio, as expected from a reaction that follows a S_N_2 mechanism (Figure 3).

Since the molar ratio LA:hexanol 1 : 6 afforded and excellent hexyl levulinate yield (98 mol %), higher hexanol ratios were not tested. The reason of that decision lies in high dilution of reactants could lead to mass‐transfer issues during reaction, and consequently affect negatively the final AL yield, as reported previously by other authors.[^22^, ^65^, ^66^]

Summarizing, the molar ratio LA:hexanol 1 : 6 was selected for further experiments since it afforded an excellent hexyl levulinate yield with a non‐detrimental increase in dihexyl ether yield.

**Catalyst type**. To corroborate the suitability of *p*‐TSA as catalyst for the MW‐assisted esterification of LA, the reaction was carried out using different catalysts for the sake of comparison. The catalysts selected were the classical mineral acid catalysts employed in esterification reactions (HCl, H_2_SO_4_ and H_3_PO_4_) and other alternative solid catalysts (Lewis acids supported on silica and acidic clays). Table 2 shows the hexyl levulinate and dihexyl ether yields for each catalyst tested under the previously optimized conditions.

**Table 2:** Dependence of hexyl levulinate and dihexyl ether yields on catalyst type. Reaction conditions: 200 °C, 5 minutes reaction time, levulinic acid (LA):hexanol molar ratio 1 : 6, catalyst loading 5 wt %.

Entry	Catalyst	Hexyl levulinate yield (mol %)	Dihexyl ether yield (mol %)
1	‐	47.49±0.45	0
2	*p*‐TSA	98.85±1.28	2.03±0.30
3	H_2_SO_4_	98.45±1.71	7.26±0.24
4	HCl	94.23±1.54	0^[a]^
5	H_3_PO_4_	64.45±1.84	0
6	Montmorillonite KSF	72.54±3.03	0.76±0.10
7	Montmorillonite K10	84.96±8.38	1.09±0.30
8	Bentonite	45.06±5.01	0.55±0.00
9	Si(Al)	38.76±1.97	0
10	Si(Ti)	56.01±1.80	0

[a] Hexyl chloride formation was observed instead of dihexyl ether.

When LA esterification is carried out in absence of catalyst (Table 2, entry 1), an acceptable 47 mol % hexyl levulinate yield was obtained in only 5 minutes, with no formation of dihexyl ether. The use of H_2_SO_4_ (Table 2, entry 3) afforded a 98 mol % hexyl levulinate yield, as excellent as that obtained with *p*‐TSA (Table 2, entry 2). However, the dihexyl ether yield when using H_2_SO_4_ as catalyst was considerably higher (7 mol %) in comparison to *p*‐TSA (2 mol %), exceeding industrially acceptable levels.^[53]^ In regard to the other two mineral acids, HCl (Table 2, entry 4) and H_3_PO_4_ (Table 2, entry 5), surprisingly the formation of dihexyl ether was not observed for any of them. For HCl, the main byproduct observed was hexyl chloride (1.5 mol % yield) and not dihexyl ether. The higher nucleophilicity of the chlorine anion (Cl^−^) compared to hexanol, makes this the predominant side reaction underwent by hexanol, instead of the formation of dihexyl ether, *via* a S_N_2 mechanism. For H_3_PO_4_, the most plausible explanation to this fact, as well as the lower hexyl levulinate yield (64 mol %) likely resides in the higher p*K_a_* of H_3_PO_4_ compared to *p*‐TSA, H_2_SO_4_ or HCl. Thus, *p*‐TSA could entail an alternative to classical mineral acids HCl, H_2_SO_4_ or H_3_PO_4_, since combines at the same time excellent hexyl levulinate yield and low dihexyl ether formation.

Regarding acidic clays, montmorillonite KSF and K10 (Table 2, entries 6–7) have been extensively used as catalyst in different organic reactions,^[67]^ as well as in esterification reactions.[^68^, ^69^, ^70^] In contrast to the main trend described in the literature, in which the higher acidity of montmorillonite KSF led to better results than montmorillonite K10,[^68^, ^69^] in this study, montmorillonite K10 afforded a higher hexyl levulinate yield than montmorillonite KSF (85 mol % *vs* 73 mol %). A plausible explanation for this observation could reside in the hydrophilicity of the solvent, that would invert the reactivity of the clays. With hydrophilic alcohols, such as glycerol, ethylene glycol^[69]^ or short‐chain alcohols (C1‐C4),^[68]^ montmorillonite KSF afforded better results. However, in this case, the longer alkyl chain of hexanol increases its hydrophobicity, and the higher reactivity of montmorillonite K10 is in accordance with the observations of Vijayakumar *et al*. who used o‐xylene (hydrophobic) as solvent, as well as low hydrophilic reagents as o‐cresol and stearic acid.^[70]^ The third acidic clay tested, Bentonite (Table 2, entry 8) afforded a low hexyl levulinate yield (45 mol %), similar to that obtained in the absence of catalyst, likely due to its lower acidity compared with the aforementioned clays. In summary, the performance of the acidic clays studied in terms of hexyl levulinate yield and reproducibility, was far from that for the *p*‐TSA‐catalyzed (Table 2, entry 2) esterification.

Finally, Lewis acid catalysts supported on silica (Table 2, entries 9–10) afforded significantly lower hexyl levulinate yields (39–56 mol % *vs* 98 mol %) than that obtained with *p*‐TSA (Table 2, entry 2).

Given the above, it was corroborated the outstanding performance of *p*‐TSA as catalyst for the MW‐assisted esterification of LA, and was selected as the best catalyst for further experiments. Additionally, the use of *p*‐TSA entails a step forward in the sustainability of the esterification of LA, since its full recovery after reaction has been already proven.[^31^, ^32^, ^33^] This positive feature of *p*‐TSA contrasts with the widely used mineral acids, for which recovery is challenging.

**Figure 4:**
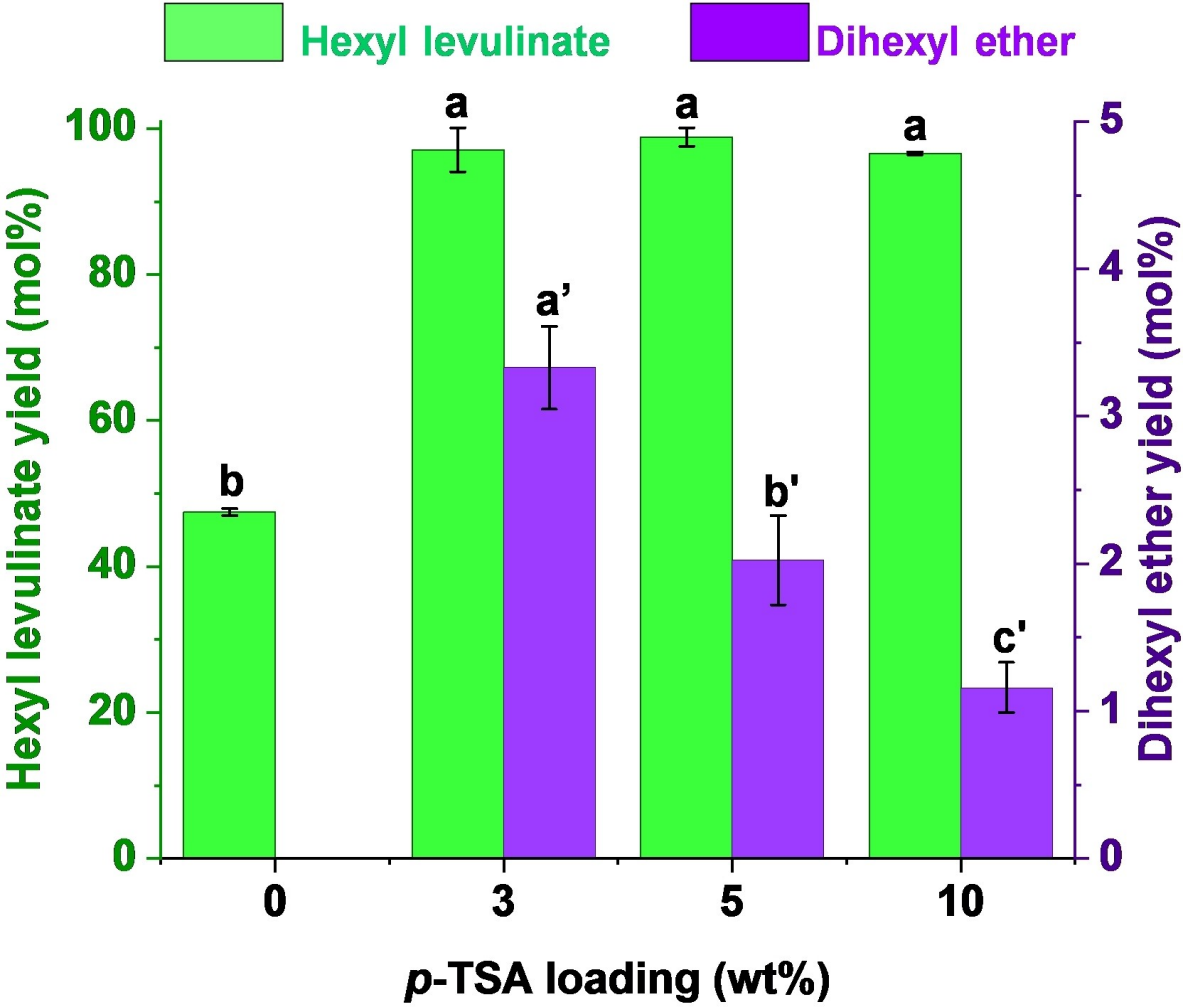
Dependence of hexyl levulinate and dihexyl ether yields on catalyst loading. Reaction conditions: 200 °C, 5 minutes reaction time, levulinic acid (LA):hexanol molar ratio 1 : 6. *Results are represented as means ± SD, n=3 and values within each variable with the different letters are significantly different (p<0.05).

**Catalyst loading**. Once *p*‐TSA was selected as the best catalyst among all tested, its loading effect was studied within a range from 0 to 10 wt %. Nguyen *et al*.^[71]^ examined the esterification of LA with ethanol under MW radiation in absence of catalyst, relying solely on the autocatalytic performance of LA, reporting a 90 % ethyl levulinate yield after 3 hours. Under the reaction conditions described in the caption of Figure 4, in the absence of *p*‐TSA, a 47 mol % hexyl levulinate yield was obtained after only 5 minutes, without formation of dihexyl ether. In presence of *p*‐TSA as catalyst (3 or 5 wt %), hexyl levulinate yield was boosted up to 98 mol %, with no significative differences between both *p*‐TSA loadings (p>0.05). However, the dihexyl ether yield was significantly lower when using 5 wt % *p*‐TSA, probably due to the fact that at higher catalyst dilution, alcohol dehydration became more favorable than esterification, since alcohol is the most abundant moiety in the reaction medium. Higher *p*‐TSA loading (10 wt %) corroborated this trend and dihexyl ether yield was significantly lower than those at lower *p*‐TSA loadings (p<0.05), while hexyl levulinate yield remained in a similar value (97 mol %). Despite achieving significantly similar hexyl levulinate yields and lower dihexyl ether formation at 10 wt % *p*‐TSA loading, it was not selected as the optimal loading. The basis of that decision resides in the green chemistry principles, since one of their goals is to reduce the usage of chemicals. Then, it is needed to reach a compromise point between achieving an acceptable amount of dihexyl ether using the lowest amount of catalyst as possible. Consequently, 5 wt % *p*‐TSA loading was selected as optimal due to involve high hexyl levulinate yield (98 mol %), low dihexyl ether yield (≤ 2 mol %) and lower catalyst usage.

## Green Chemistry Assessment

**Recovery of unreacted hexanol**. To align the synthetic procedure described in this work with the principles of green chemistry and SDGs, a circular loop to recover and reuse the excess of unreacted hexanol was envisaged. The feasibility of this approach was initially assessed by investigating the recovery of unreacted hexanol through simple distillation. Subsequently, its reusability in LA esterification was examined to ensure that high yields of hexyl levulinate could still be achieved with low dihexyl ether formation.

For this purpose, MW‐assisted esterification of LA with hexanol was carried out on a larger scale than in previous experiments, as described in the experimental section. In total, 88 wt % of the unreacted hexanol was recovered through simple distillation with optimal purity, as confirmed by the ^1^H NMR spectrum (Figures S7‐S8). The recovered hexanol was then submitted to MW‐assisted esterification of LA again. The reused hexanol yielded hexyl levulinate at 95.21±0.51 mol %, with a dihexyl ether yield of only 1.22±0.09 mol %. This demonstrated the feasibility of the proposed circular loop for the sustainable reuse of resources.

**Figure 5:**
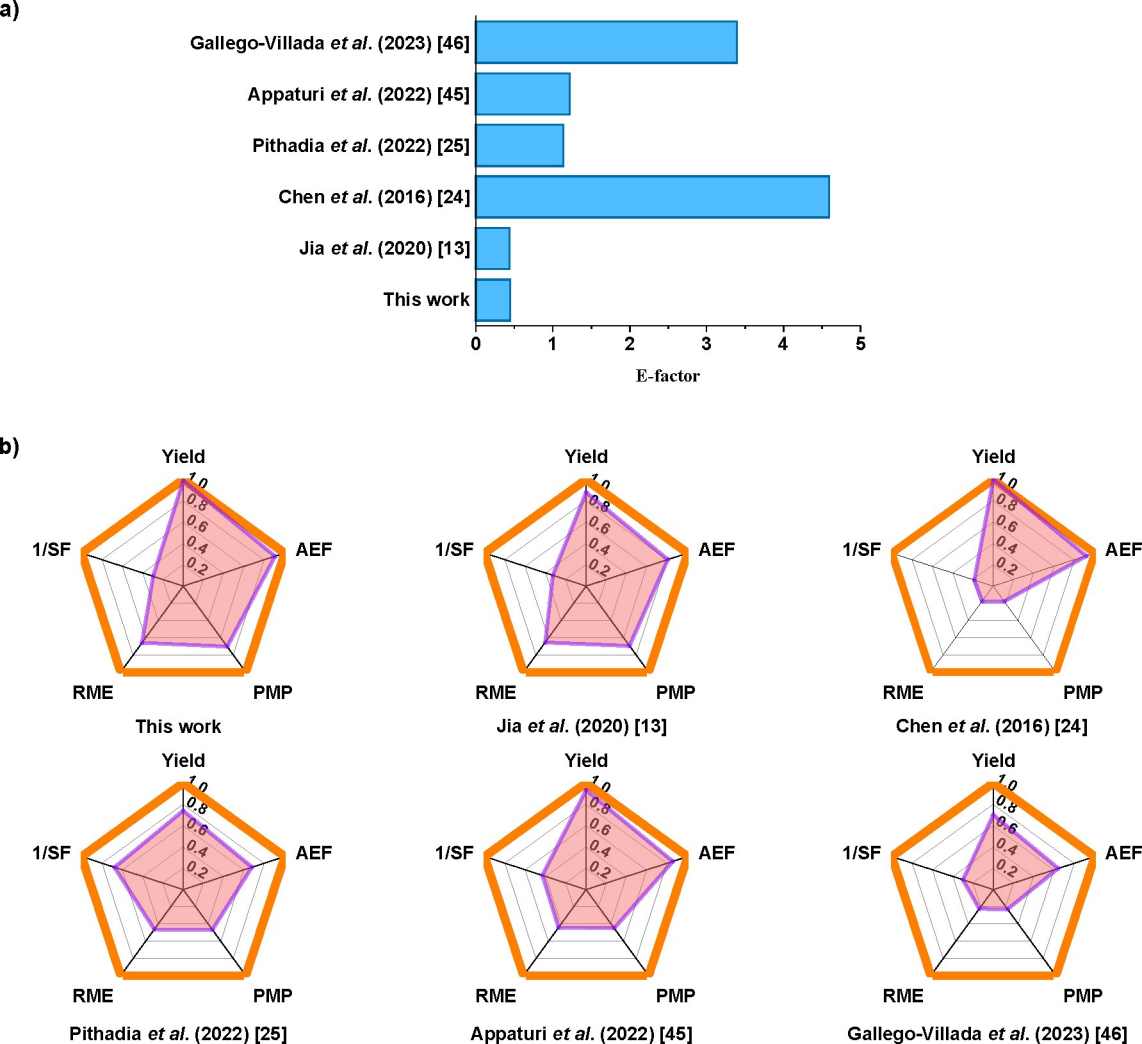
Comparison of this work with the related literature on E‐factor a), and yield, atom efficiency (AEF), process mass productivity (PMP), reaction mass efficiency (RME) and 1/stoichiometric factor (1/SF) b) for the levulinic acid esterification with hexanol. The orange line denotes optimal values.

**Evaluation of Green Chemistry Performance Metrics (GCPMs)**. In order to assess the environmental benefits of this method, and to stablish a valid comparison with the related literature, several GCPMs for the esterification of LA with hexanol have been calculated as described in the supporting information (Equations S2–S8 and Table S2). A comparison of the GCPMs studied between this work with the related literature is gathered in Figure 5. Notably, due to the feasibility of recycling the unreacted hexanol, the process herein described achieved an excellent E‐factor score of 0.44. This value is in line with the work of Jia *et al*. (E‐factor =0.44) (Figure 5a) who also stated the recycling of the unreacted hexanol.^[13]^ When the E‐factor is calculated for processes that do not recycle the unreacted hexanol,[^24^, ^25^, ^45^, ^46^] this is substantially higher (E‐factor >1) compared to the value obtained in this work. These findings underscore the significant impact of recycling the excess solvent on the environmental footprint of the process.

Regarding other GCPMs, in Figure 5b could be observed the outstanding hexyl levulinate yield achieved (98 mol %) placed this work among the best regarding the AEF value. This metric expresses the AE, but taking into account the yield of the desired product. It was obtained an AEF=90.69 %, at the same level of the work of Chen *et al*. (AEF=91.75 %),^[24]^ but with a much shorter reaction time (5 minutes *vs* 10 hours, Table 1, entry 14 *vs* entry 2) and lower catalyst loading (5 *vs* 25 wt %, Table 1, entry 14 *vs* entry 2). This metric corroborates the relevance of achieving high yields (>90 %) in developing more sustainable and green chemical process. However, due to the nature of esterification reaction, which needs an excess of alcohol to achieve high ester yields, the metric related to the stoichiometry of the process (SF) afforded modest results (SF=3.36). In this regard, the studies that used lower excess of hexanol scored better values in this metrics (Figure 5b), but at the expense of hexyl levulinate yield (74 %, Table 1, entry 3)^[25]^ or by using high catalyst loading (69 wt %, Table 1, entry 10).^[45]^

Although the score for SF might initially appear as drawback for this work, the successful recovery of most of the unreacted hexanol mitigated this issue by significantly reducing waste formation, as evidenced by the good PMP and RME values obtained (PMP=70.23 %; RME=65.78 %). Following the same trend as E‐factor commented above, the recovery of excess reagents or solvents has a positive impact in the GCPMs, since this work and the work of Jia *et al*. achieved the best scores for PMP and RME (Figure 5b)

Additionally, the evaluation of the Eco‐score also provided an excellent evaluation in terms of safety and feasibility for this work. According to the penalty points described by Van Aken *et al*., this method received 12.575 penalty points, as detailed in the supporting information (Table S2). Therefore, this process scored 87.425 on the eco‐scale, which is considered excellent according to the developers of this metric.^[72]^

Globally, the assessment of the GCPMs corroborated the sustainability of this process. These metrics, combined with the excellent AL yield achieved, the short reaction time resulting in energy savings, and the low catalyst loading entail a step forward in the field compared to the existing literature.

**Reaction scope**. Finally, to evaluate the versatility of the esterification method developed in this work, the reaction parameters that afforded the highest hexyl levulinate yields and the lowest formation of dihexyl ether, were applied to the LA esterification with other alcohols. Developing synthetic methods that could be easily adapted to different substrates plays a key role for their future deployment at industrial scale.^[25]^ Table 3 presents the results obtained, illustrating the influence of alcohol length (from 2 to 10 carbon atoms) and type (primary and linear, primary and branched, secondary and linear, and secondary and cyclic) on the yields of AL and dialkyl ether. This comprehensive assessment allows us to determine the adaptability of this esterification method across various alcohol substrates, providing valuable insights for potential industrial applications.

**Table 3:** Dependence of alkyl levulinate and dialkyl ether yield depending on the type of alcohol. Reaction conditions: 200 °C, 5 minutes reaction time, levulinic acid (LA):alkyl alcohol molar ratio 1 : 6, *p*‐toluenesulfonic acid (*p*‐TSA) 5 wt % as catalyst.

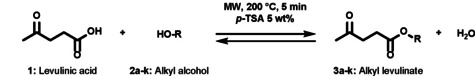						
Entry	Alcohol	2	Product	3	Levulinate yield (mol %)	Dialkyl ether yield (mol %)
1	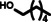	2a	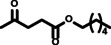	3a	98.85±1.28	2.03±0.30
2	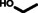	2b	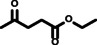	3b	90.81±0.44	1.07±0.08
3	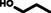	2c	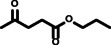	3c	93.31±0.50	0.69±0.11
4	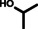	2d	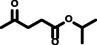	3d	88.92±0.74	2.45±0.11
5	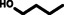	2e	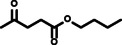	3e	94.01±0.47	2.53±0.16
6	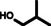	2f	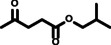	3f	92.43±0.33	<0.5
7	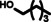	2 g	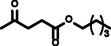	3 g	96.88±2.32	1.81±0.25
8	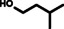	2 h	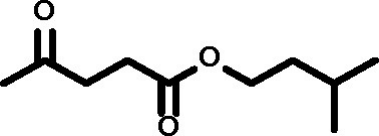	3 h	97.11±2.33	1.69±0.23
9	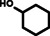	2i	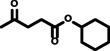	3i	92.07±0.26	n. d.^[a]^
10	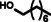	2j	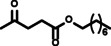	3j	97.25±2.84	1.56±0.05
11	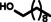	2k	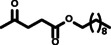	3k	98.60±0.22	0.70±0.03

n.d.: not detected. [a] Cyclohexene formation was observed instead of dicyclohexyl ether

**Figure 6:**
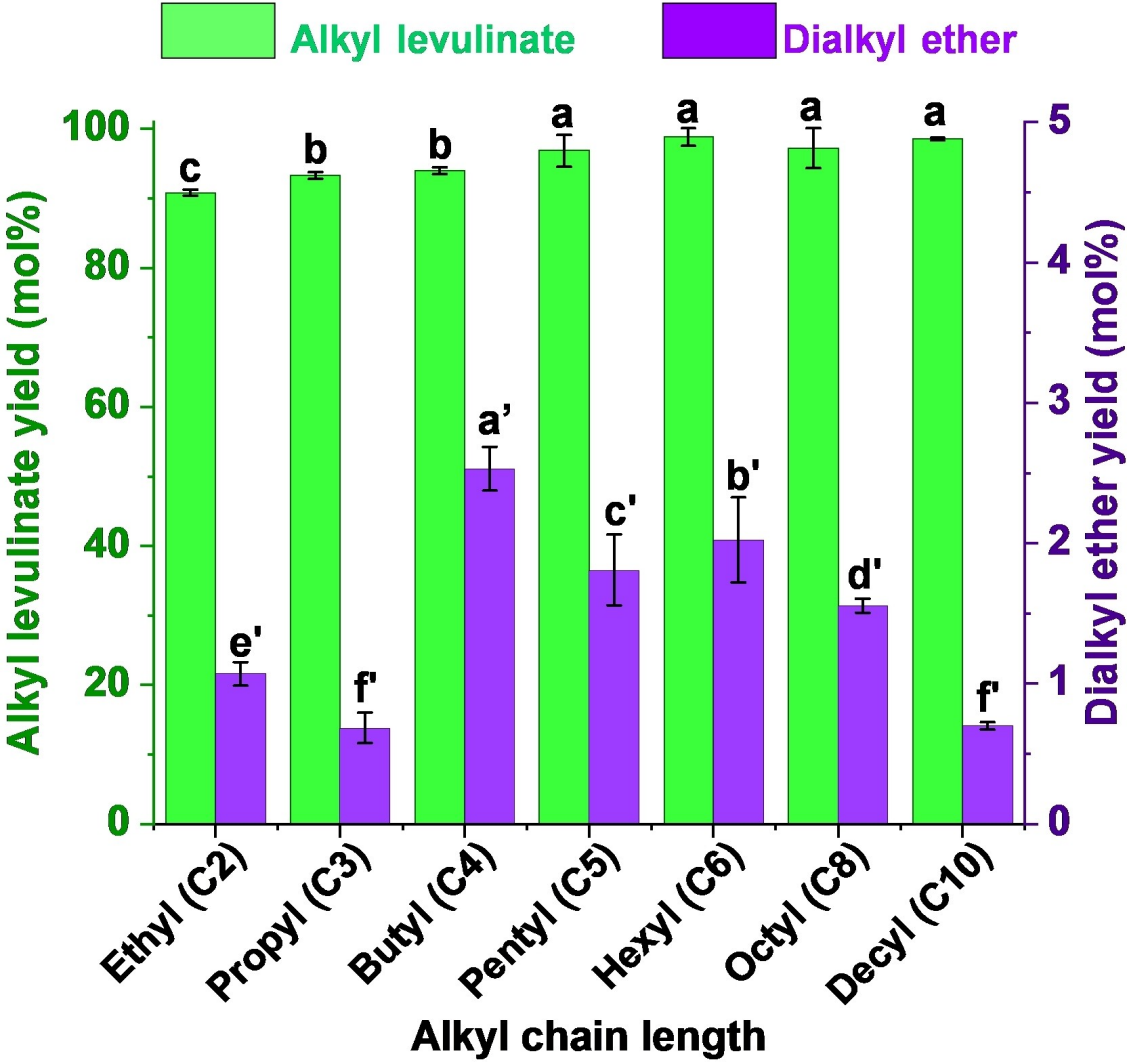
Dependence of alkyl levulinate and dialkyl ether yields on alkyl chain length of primary and linear alcohols. Reaction conditions: 200 °C, 5 minutes reaction time, levulinic acid (LA):alkyl alcohol molar ratio 1 : 6, *p*‐toluenesulfonic acid (*p*‐TSA) 5 wt% as catalyst. *Results are represented as means ± SD, n=3 and values within each variable with the different letters are significantly different (p<0.05).

In light of the results obtained for primary and linear alcohols (Table 3 and Figure 6), the utility of this method for synthesizing medium‐ and long‐chain Als could be highlighted. Octanol (Table 3, entry 10) and decanol (Table 3, entry 11) afforded the highest AL yields, as well as the lowest dialkyl ether yields. These high yields for long‐chain ALs provide strong support to this method, since it allows us to overcome the lower nucleophilicity of long chain alcohols, which typically inhibit their ability to donate electron pairs to the carbonyl atom of LA, making them less reactive, as stated by other authors.^[46]^ Figure 6 clearly depicts an increasing trend in AL yields from ethanol (C2) to decanol (C10) (p<0.05). Conversely, dialkyl ether yields followed the opposite trend, decreasing from butanol (C4) to decanol (C10) (p<0.05). The low dialkyl ether yields (≤ 1 mol %) for ethanol (C2) and propanol (C3) seem to contradict the aforementioned statement. However, this disparity is likely attributed to the low boiling point of these ethers (diethyl ether bp: 35 °C; dipropyl ether bp: 90 °C), leading to their rapid evaporation and underestimation of their formation extent.

Regarding primary and branched alcohols, 2‐methyl‐1‐propanol (Table 3, entry 6) and 3‐methyl‐1‐butanol (Table 3, entry 8), it was observed that the proximity of the branch to the hydroxyl group influences both AL and dialkyl ether yields. For ALs, the yield of 3‐methyl‐1‐butyl levulinate (Table 3, entry 8) was 97 mol %, but when the ramification is closer to the hydroxyl group, as happens for 2‐methyl‐1‐propyl levulinate, the yield dropped to 92 mol %. The same observation occurred for dialkyl ether yields, since it dropped from 1.69 mol % (Table 3, entry 8) to <0.5 mol % (Table 3, entry 6). Extending the comparison of primary and branched alcohols with their corresponding primary and linear counterparts (butanol for 2‐methyl‐1‐propanol and pentanol for 3‐methyl‐1‐butanol) revealed the following: Between pentanol (Table 3, entry 7) and 3‐methyl‐1‐butanol (Table 3, entry 8) the yields for both ALs (97 *vs* 97 mol %) and dialkyl ethers (1.81 *vs* 1.69 mol %) remained constant. However, when comparing the yields using butanol (Table 3, entry 5) or 2‐methyl‐1‐propanol (Table 3, entry 6) a slight decrease in AL yield was observed (94 *vs* 92 mol %), while a strong drop in dialkyl ether formation happened (2.53 *vs* <0.5 mol %). The most plausible explanation for these differences in AL and dialkyl ether yields is probably due to the steric hinderance of the hydroxy group.^[25]^

Additionally, two secondary alcohols were included in the reaction scope to assess the performance of this method when the esterification reaction competes with subtracts that can also undergo elimination reactions. The alcohols selected for this purpose were 2‐propanol (Table 3, entry 4) and cyclohexanol (Table 3, entry 9). In both cases, the AL yields (89 mol % for 2‐propyl levulinate, 92 mol % for cyclohexyl levulinate) were lower than those obtained for primary alcohols, but still excellent. It is worth mentioning the remarkable yield of cyclohexyl levulinate obtained (92 mol %) since it is the most sterically hindered compound tested. Finally, as commented above, secondary substrates are also prone to undergo elimination reactions in acidic media to give alkenes. Then, the extent of the elimination reaction was also assessed. For 2‐propanol, the elimination product (propylene) was not detected, probably due to the low boiling point of propylene (−47 °C). However, the dialkyl ether yield was in the range of other substrates (~2 mol %). Interestingly, the formation of dicyclohexyl ether was not detected. Instead, the elimination product (cyclohexene) was present in a 9 mol % yield.

## Conclusions

Aligned with the current demand for implementing more sustainable processes for energy and fuel production, a MW‐assisted method for synthesizing ALs from biomass‐derived LA has been developed, since they are promising biofuel and fuel additives. Through a careful investigation of various reaction parameters on the MW‐assisted esterification of LA with hexanol, an excellent 98 mol % hexyl levulinate yield was obtained in just 5 minutes at 200 °C, using a LA:hexanol molar ratio of 1 : 6 and 5 wt % *p*‐TSA as catalyst. In addition, the formation of undesirable dialkyl ether by‐products was minimized to acceptable industrial levels (~2 mol % yield), and no LA aldol condensations or acetal products were observed. The high yields obtained, together with the use of an economically affordable catalyst and the low side‐product formation, provides a strong support for a future scaling‐up of this process. Furthermore, in the pursuit of achieving sustainability and circularity, 88 wt % of the unreacted hexanol was successfully recovered through simple distillation, demonstrating the feasibility of reusing unreacted hexanol in subsequent LA esterification with comparable results to fresh hexanol.

The method developed has exhibited versatility when other alcohols were used, affording excellent AL yields (≥89 mol %) with limited dialkyl ether formation (≤2.5 mol %). Notably, exceptionally high yields for long‐chain Als were achieved. For instance, a 99 mol % yield was afforded for decyl levulinate, overcoming the expected lower reactivity associated with longer alkyl chains.

Collectively, the assessment of the GCPMs and eco‐scale for this process corroborated that this work could pave the way for the deployment of greener and energy‐efficient synthetic methods for obtaining medium‐ and long‐chain ALs. This could set a step forward in the transition from fossil‐carbon fuels to bio‐based and renewable alternatives.

## Experimental Section

### Materials

All chemicals employed with the exception of Lewis acids supported on silica were available commercially and used without further purification steps. The suppliers and purity of the chemicals employed are detailed in the supporting information. Lewis acid catalysts supported on silica Si(Al) and Si(Ti) synthesis is described in the supporting information (Scheme S1).

### MW‐Assisted Esterification Reaction

Esterification reactions were performed using a CEM Discover SP monomode MW reactor (CEM Discover SP, Matthews, NC, USA) in a sealed vessel at a temperature of 200 °C for a selected duration (0–90 minutes), following this procedure: Initially, 2 ml of alcohol and the appropriate amounts of acidic catalyst and LA were added to a 10 mL MW vessel containing a stirring bar, and then stirred for homogenization. Next, the vessel was tightly sealed, placed into the MW reactor and subjected to the aforementioned conditions. The maximum allowable power and pressure were set at 180 W and 250 psi, respectively. Heating, power and pressure profiles of a representative reaction are shown in Figure S1. Each reaction was conducted by triplicate, and the reported values represent the averages.

### Nuclear Magnetic Resonance (NMR) Analysis

NMR spectra were recorded at 25 °C using a Bruker Ascend™500 spectrometer (Bruker Corporation, Billerica, MA, USA) operating at a frequency of 500.16 MHz for the ^1^H nucleus and 125.77 MHz for ^13^C. CDCl_3_ was employed as solvent and chemical shifts were referenced to tetramethylsilane (TMS) at 0 ppm.

For quantitative purposes, a 10 μL aliquot of the crude reaction mixture was placed in a 5 mm NMR tube and dissolved in 500 μL of CDCl_3_. Then, ^1^H NMR spectra were recorded using the following acquisition parameters: spectral width 7142.86 Hz, acquisition time 4.59 s, 16 scans, 90° pulse width of 8 μs and relaxation delay 25 s. All spectra were processed using the TopSpin 3.6.2. software (Bruker Biospin Gmbh, Rheinstteten, Germany). Calculations are described in the supporting information using Equation S1.

### Product Purification

To remove possible unreacted LA and the *p*‐TSA, the crude reaction mixture was extracted with saturated NaHCO_3_ (2 x 2 mL). Subsequently, vacuum distillation was employed to recover the alkyl levulinate and any excess unreacted alcohol from the organic phase.

### Scaling‐Up and Unreacted Hexanol Recovery

MW‐assisted esterification of LA was carried out in a 35 mL vessel following the protocol described previously: 10 mL of hexanol (8.21 g, 80.35 mmol) was added to the vessel together with 1.56 g (13.39 mmol) of LA and 77.75 mg (5 wt %) of *p*‐TSA. The reaction conditions were as previously optimized. After reaction, the crude mixture was washed with saturated NaHCO_3_ (2 x 10 mL) to remove acids and then distilled to recover the unreacted hexanol. The recovered hexanol was submitted again to MW‐assisted LA esterification under the optimized conditions following the procedure described previously.

### GCPMs for Hexyl Levulinate Synthesis

To assess the environmental impact of the methodology developed in this work, several GCPMs have been calculated for the scaled‐up esterification of LA with hexanol under microwave radiation. The evaluated GCPMs were environmental factor (E‐factor), atom economy (AE), atom efficiency (AEF), process mass intensity (PMI), process mass productivity (PMP), reaction mass efficiency (RME), stoichiometric factor (SF) and eco‐scale. The description of each metric and their calculation are described in the supporting information (Equations S2–S8 and Tables S1‐S2).

### Statistical Analysis

Experimental data were analyzed using the one‐way analysis of variance (ANOVA). The significant difference among the mean values was examined by Duncan′s test (P≤0.05) using the SPSS software (ver. 23.0).

## Supporting Information Summary

Supporting Information includes the following information: purity and suppliers of chemicals employed; experimental protocol for synthesizing Si(Al) and Si(Ti) catalysts; heating, power and pressure profiles of MW‐assisted reaction; qNMR and green chemistry metrics calculations; side reactions assessment; hexanol recovery; ^1^H NMR and ^13^C{^1^H} NMR spectra of alkyl levulinates synthesized (Figures S9–S30). The authors have cited additional references within the Supporting Information.[^73^, ^74^, ^75^, ^76^, ^77^]

## Author Contributions

Alberto J. Huertas‐Alonso: Methodology, Validation, Formal Analysis, Investigation, Writing – Original Draft preparation, Writing – Review & Editing. Diego J. González‐Serrano: Methodology, Formal Analysis, Investigation, Writing – Original Draft preparation. Manuel Salgado‐Ramos: Validation, Formal Analysis, Writing – Review & Editing. Milad Hadidi: Validation, Formal Analysis, Writing – Review & Editing, Visualization. Mª Prado Sánchez‐Verdú: Conceptualization, Writing – Review & Editing, Supervision, Funding Acquisition. Beatriz Cabañas: Resources, Supervision, Funding acquisition. Christopher J. Chuck: Writing – Review & Editing, Visualization. James H. Clark: Writing – Review & Editing, Visualization. Andrés Moreno: Conceptualization, Resources, Writing – Review & Editing, Supervision, Project administration, Funding acquisition.

## Conflict of Interests

The authors declare no conflict of interest.

1

## Supporting information

As a service to our authors and readers, this journal provides supporting information supplied by the authors. Such materials are peer reviewed and may be re‐organized for online delivery, but are not copy‐edited or typeset. Technical support issues arising from supporting information (other than missing files) should be addressed to the authors.

Supporting Information

## Data Availability

The data that support the findings of this study are available in the supplementary material of this article.
