# Supplementary material for: Sustainable Microwave‐Assisted Synthesis of Medium‐ and Long‐Chain Alkyl Levulinates from Biomass‐Derived Levulinic Acid
Source: ChemSusChem. 2025 Mar 17;18(10):e202402508. doi: 10.1002/cssc.202402508 (PMC12094142; doi:10.1002/cssc.202402508)
Supplement: Supplementary file 1 — Supporting Information [file CSSC-18-e202402508-s001.pdf]

ChemSusChem

Supporting Information

Sustainable Microwave-Assisted Synthesis of Medium- and Long-Chain Alkyl Levulinates from Biomass-Derived Levulinic Acid

Alberto J. Huertas-Alonso,* Diego J. González-Serrano, Manuel Salgado-Ramos, Milad Hadidi, Prado Sánchez-Verdú, Beatriz Cabañas, Christopher J. Chuck, James H. Clark, and Andrés Moreno*

Supporting information for

Sustainable Microwave-assisted Synthesis of Medium- and Long-chain Alkyl Levulinates from Biomass-derived Levulinic Acid

Alberto J. Huertas-Alonso[1,2,†‡]*, Diego J. González-Serrano[1‡], Manuel Salgado-Ramos[1], Milad Hadidi[1], Mª Prado Sánchez-Verdú[1], Beatriz Cabañas[3], Christopher J. Chuck[4], James H. Clark[5], Andrés Moreno[1*]

[1] Universidad de Castilla-la Mancha, Departamento de Química Inorgánica, Orgánica y Bioquímica, Facultad de Ciencias y Tecnologías Químicas, Avenida Camilo José Cela nº10, 13005 Ciudad Real, Spain.

[2] Universidad de Castilla-La Mancha, Departamento de Química Física, Facultad de Ciencias y Tecnologías Químicas, Avenida Camilo José Cela s/n, 13071 Ciudad Real, Spain.

[3] Universidad de Castilla-La Mancha, Instituto de Combustión y Contaminación Atmosférica (ICCA), Camino de Moledores s/n, Ciudad Real 13005, Spain.

[4] Department of Chemical Engineering, University of Bath, Bath, BA2 7AY, United Kingdom.

[5] Green Chemistry Centre of Excellence, University of York, Department of Chemistry, Heslington, York, YO10 5DD, UK.

[‡] Contributed equally

[†] Present address: Department of Materials and Environmental Chemistry, Stockholm University, SE-106 91 Stockholm, Sweden.

* Correspondence should be addressed to:

Dr. Alberto J. Huertas-Alonso, albertojose.huertasalonso@mmk.su.se

Prof. Andrés Moreno, andres.moreno@uclm.es

Table of contents

1. **Materials and methods**
2. **Quantitative Nuclear Magnetic Resonance (qNMR) calculations**
3. **Green chemistry performance metrics (GCPMs)**
4. **Side reactions assessment**
5. **Hexanol recovery**
6. **^{1}H NMR and ^{13}C{^{1}H} NMR spectra of the products**
7. **References**

1. Materials and methods

Levulinic acid (≥ 98 %) was purchased from Roth. Ethanol absolute (≥ 99.8 %), 1-butanol (≥ 99.5 %), 2-methyl-1-propanol (99 %), 1-pentanol (98 %), 3-methyl-1-butanol (98.5 %), $NaHCO_3$ (≥ 99.7 %), silica gel (3-6 mm), HCl (37 %) and H_3PO_4 (85 %) were purchased from Panreac. 1-Propanol (≥ 99.5 %), 1-hexanol (≥ 98 %), *p*-toluenesulfonic acid monohydrate (≥ 98.5 %) and Titanium (IV) chloride 1 M solution in toluene were purchased from Sigma-Aldrich. Cyclohexanol (98 %), 1-octanol (98 %), 1-decanol (99 %), 2-butanone (≥ 99 %) and diethylaluminium chloride 0.9 M solution in toluene were purchased from Acros Organics. 2-Propanol (≥ 99.5 %), H_2SO_4 (95 %), toluene and $CDCl_3$ (≥ 99.8 % deuterium labelling + 0.03 vol.% TMS) were purchased from VWR. Montmorillonite KSF and Bentonite were purchased from Aldrich. Montmorillonite K10 was purchased from Fluka.

Supported catalysts Si(Al) and Si(Ti) synthesis

Si(Al) and Si(Ti) were synthesized following the procedure described by Cativiela *et al.*[73] Initially, 1 g of silica gel was activated at 120 °C for 12 hours in an oven, and then at 140 °C for 2 hours under vacuum. Then, activated silica gel was transferred to a round-bottom flask along with 15 mL of anhydrous toluene. Subsequently, under argon atmosphere, 2.5 mmol of the corresponding Lewis acid from commercially available solutions in toluene (Titanium chloride (IV) or diethyl aluminum chloride) was added to the round-bottom flask and heated to reflux for 48 hours. Finally, solvent was removed, and the catalyst supported on silica rinsed with anhydrous toluene. Si(Al) was obtained as a brownish-orange color powder, while Si(Ti) was obtained as a dark brown/black color powder.

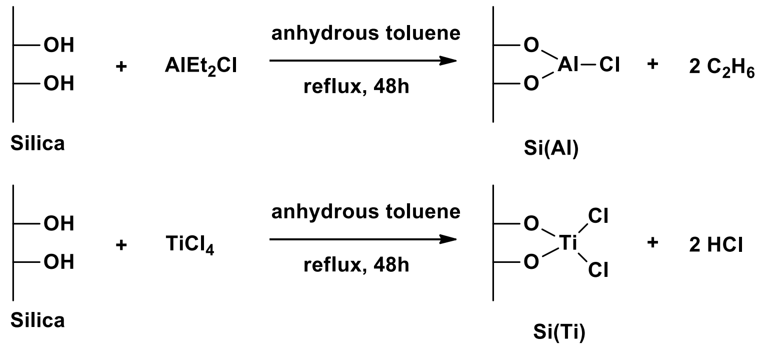

Scheme S1. Supported catalysts synthesis. Adapted from reference [73].

Heating, power and pressure profile of MW-assisted esterification

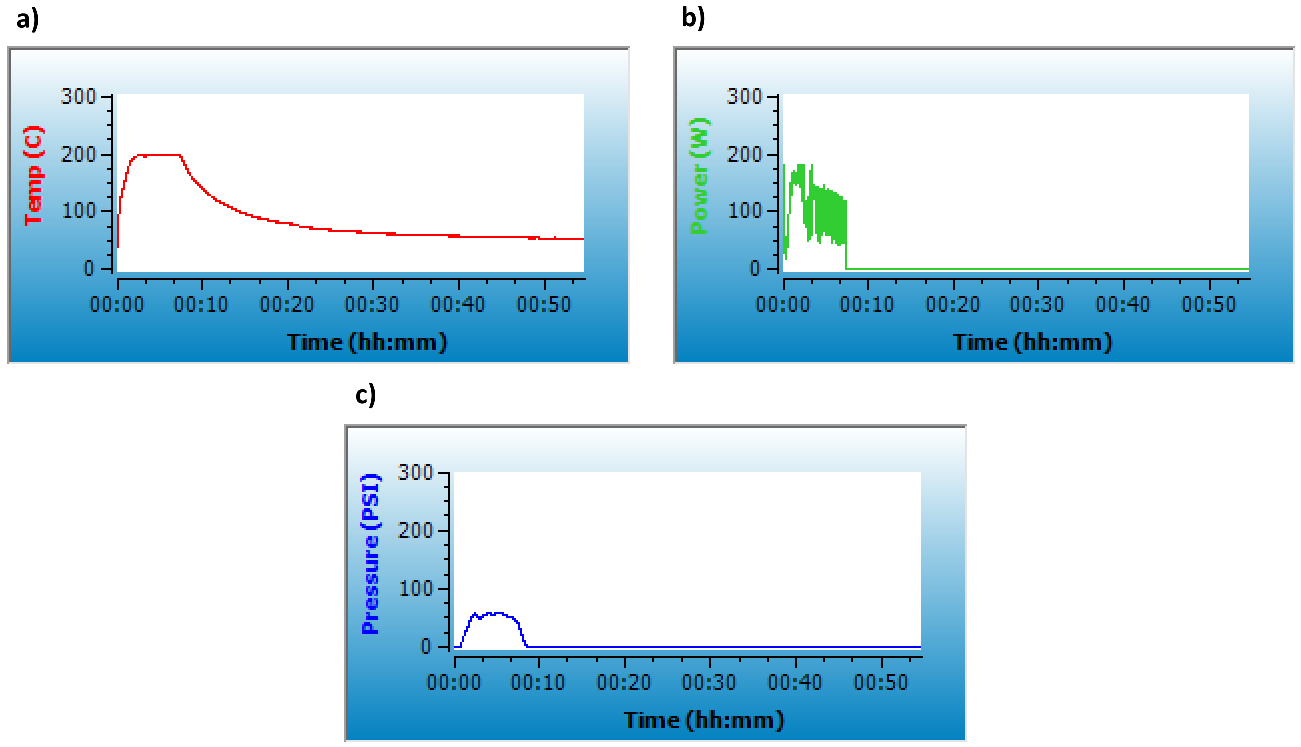

Figure S1. Heating **a)**, power **b)** and pressure **c)** profiles for the MW-assisted esterification of levulinic acid (LA) with hexanol. Reaction conditions: 200 °C, 5 min reaction time, LA:hexanol molar ratio 1:6, *p*-toluenesulfonic acid (*p*-TSA) 5 wt.% as catalyst.

2. Quantitative Nuclear Magnetic Resonance (qNMR) calculations

Due to levulinic acid only undergoes esterification under the conditions described in this work, and no other by-products were observed, the alkyl levulinate yields and alcohol conversions into the corresponding dialkyl ether were calculated by means of qNMR, using the relative method described by Holzgrabe[60] shown in **Equation S1**:

$$\frac{nx}{ny} = \frac{Ix}{Iy} * \frac{Ny}{Nx}$$

Equation S1

Where n_x/n_y is the molar ratio of compounds *x* and *y*, *I* is the value of the signal integral and *N* the number of contributing nuclei to the signal considered (**Figure S2**). Since no levulinic acid auto condensation products were detected, as commented in the previous section, it was assumed that the only product obtained from levulinic acid was the corresponding levulinate ester. Thus, levulinate yield could be expressed as the molar ratio calculated using **Equation S1** between the integral selected for the alkyl levulinate (a’) and the sum of the signals selected for levulinic acid and alkyl levulinate (a + a’’). For dialkyl ether yields (a’’’’), the sum of signal considered were a’ + a’’’ + a’’’’.

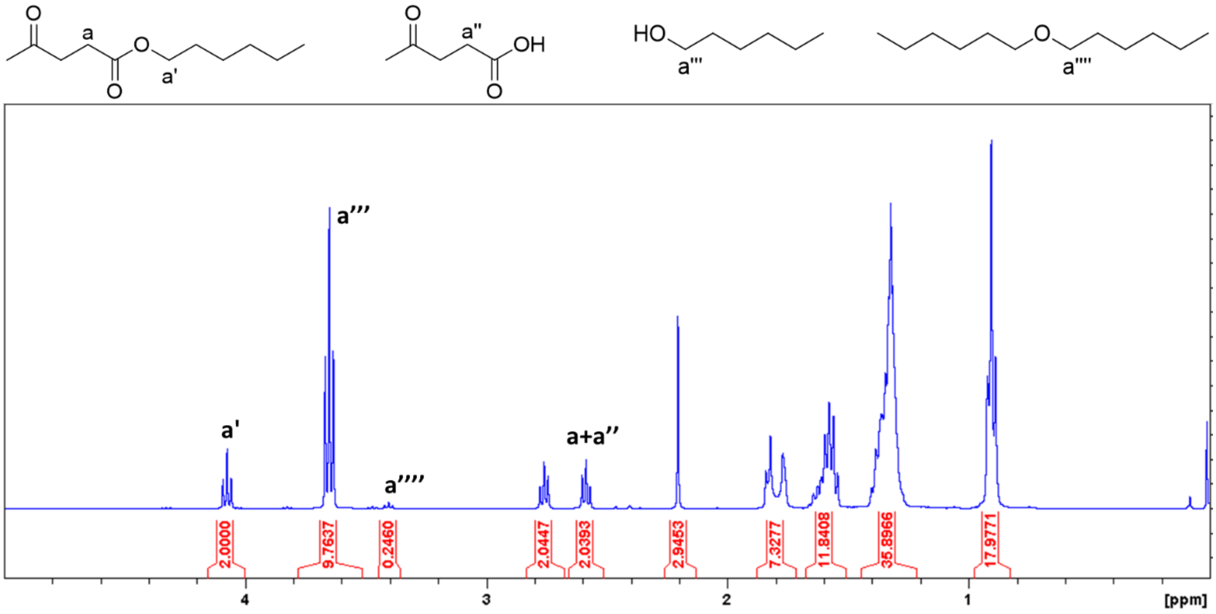

Figure S2. ^{1}H NMR spectrum (500 MHz, $CDCl_3$) of a reaction crude.

3. Green chemistry performance metrics (GCPMs)

The following GCPMs were calculated as described by Salgado-Ramos *et al*.[75] and Fantozzi *et al*.[76] Eco-scale calculation was performed as described by Van Aken *et al*.[72]

For the calculation of the following GCPMs, an esterification reaction of levulinic acid with hexanol in a bigger scale was performed. The reaction conditions were the following: 200 °C, 5 minutes reaction time, molar ratio LA:Hexanol 1:6, *p*-TSA 5 wt.%. The amount of reagents used and product obtained in this reaction are collected in **Table S1**.

Table S1. Amounts of reactants and products of the esterification reaction considered for the calculation of the GCPMs.

Reactants	Amount (g)	Products	Amount (g)
Levulinic acid	1.56	Hexyl levulinate	2.65
1-Hexanol	8.21	Dihexyl ether	0.30
p-Toluenesulfonic acid	0.08		

- **Environmental factor (E-factor)**: E-factor is considered as *a posteriori* metric, that means it can be only calculated after carrying out the reaction or process. Relates to the amount of waste generated in a chemical process with respect to the amount of target compound obtained. Therefore, the desired value of this metric is as close as possible to 0.

$$E - factor\ \left(\frac{kg}{kg}\right) = \frac{Mass\ of\ waste\ (kg)}{Mass\ of\ desired\ product\ (kg)}$$

Equation S2

Usually, E-factor calculation excludes solvents and water. Additionally, chemicals that can be readily recovered could be also excluded. Esterification reaction produces water

and therefore, the water formed is excluded from the calculations. Regarding hexanol, it is also a reagent and then, the unreacted hexanol has been considered as waste unless its recovery is explicitly stated. Even though *p*-TSA could be recovered, it has been included as a waste, since its recovery has not been explored in this work.

This work

Mass of waste: non-recovered unreacted hexanol (0.78 g), unreacted levulinic acid (0.02 g), dihexyl ether (0.30 g), *p*-toluenesulfonic acid (0.08 g).

Mass of desired product: hexyl levulinate (2.65 g).

$$\boldsymbol{E-factor\left(\frac{g}{g}\right)} = \frac{0.78\,g + 0.02\,g + 0.30\,g + 0.08\,g}{2.65\,g} = \mathbf{0.44}$$

- **Atom economy (AE)**: This metric is directly related to the 2nd green chemistry principle. In contrast to E-factor, AE is considered as an *a priori* metric, that can be calculated before carrying out any experiment. AE provides the number of atoms of reactants that end up in the final product. Consequently, an AE = 100 % means that all atoms in the reactants end up in the desired product.

$$AE\ (\%) = \frac{Molecular\ weight\ of\ the\ desired\ product\ (\frac{kg}{kmol})}{Molecular\ weight\ of\ reactants\ (\frac{kg}{kmol})}\ x\ 100$$

Equation S3

This work

Desired product molecular weight: hexyl levulinate (200.27 g/mol).

Reactants molecular weight: hexanol (102.18 g/mol), levulinic acid (116.11 g/mol).

$$\boldsymbol{AE}\ (\%) = \frac{200.27\ g/mol}{102.18\ g/mol + 116.11\ g/mol}\ x\ 100 = \mathbf{91.75}$$

- **Atom efficiency (AEF)**: This metric considers the desired product yield to calculate the AE. As same as AE, AEF = 100 % is the desired value for this metric.

$$AEF\ (\%) = AE\ x\ Hexyl\ levulinate\ yield\ (\%)/100$$

Equation S4

This work

AE: 91.75 %

Hexyl levulinate yield: 98.85 %

$$\boldsymbol{AEF}\ (\%) = 91.75\ x \frac{98.85}{100} = \mathbf{90.69}$$

- **Process mass intensity (PMI)**: PMI is defined as the total mass of raw materials needed to obtain 1 kg of desired product. This metric is directly related to E-factor (PMI = E-Factor +1) and its ideal value is 1. The lower the PMI, the "greener" the process.

$$PMI\ \left(\frac{kg}{kg}\right) = \frac{Total\ mass\ used\ in\ the\ process\ (kg)}{Mass\ of\ desired\ product\ (kg)} \approx E - factor + 1$$

Equation S5

This work

Total mass used in the process: hexanol consumed (excluding recovered hexanol) (2.14 g), levulinic acid (1.56 g), *p*-toluenesulfonic acid (0.08 g).

Mass of desired product: hexyl levulinate (2.65 g).

$$\boldsymbol{PMI}\ \left(\frac{\boldsymbol{g}}{\boldsymbol{g}}\right) = \frac{2.14\ g + 1.56\ g + 0.08\ g}{2.65\ g} = \mathbf{1.42} \approx E - factor + 1$$

- **Process mass productivity (PMP)**: This metric is used to express PMI as a percentage value. Since the optimum value for PMI is 1, for PMP as percentage is 100 %.

$$PMP\ (\%) = \frac{1}{PMI}\ x\ 100$$

Equation S6

This work

$$\boldsymbol{PMP}\ (\%) = \frac{1}{1.42}\ x\ 100 = \mathbf{70.23}$$

- **Reaction mass efficiency (RME)**: This metric expresses the mass of desired product obtained (in kg) with respect to the mass of all reactants used in the process. It is related to AE, so it considers the mass of reagents consumed to yield the desired product. As percentage, the optimal value is 100 %, which means that all the reactants mass ends up in the desired product.

$$RME\ (\%) = \frac{Mass\ of\ desired\ product\ (kg)}{Mass\ of\ reactants - mass\ recovered\ (kg)}\ x\ 100$$

Equation S7

This work

Mass of desired product: hexyl levulinate (2.65 g).

Mass of reactants: hexanol (total - recovered) (2.47 g), levulinic acid (1.56 g).

$$\boldsymbol{RME}\ (\%) = \frac{2.65\ g}{2.47\ g + 1.56\ g}\ x\ 100 = \mathbf{65.78}$$

- **Stoichiometric factor (SF)**: SF considers the excess of reactants used in a process. Stoichiometric reactions have an SF = 1, while when a reagent is used in excess, SF > 1.

$$SF = 1 + \frac{Total\ mass\ of\ excess\ reagents\ (kg)}{Total\ mass\ of\ stoichiometric\ reagents\ (kg)}$$

Equation S8

This work

Total mass of excess reagents: excess hexanol (6.84 g), *p*-toluenesulfonic acid (0.08 g).

Total mass of stoichiometric reagents: stoichiometric hexanol (1.37 g), levulinic acid (1.56 g).

$$\boldsymbol{SF} = 1 + \frac{6.84 + 0.08}{1.37 + 1.56} = \mathbf{3.36}$$

- **Eco scale:** This metric was introduced by Van Aken *et al*. to provide a qualitative assessment of an organic reaction by evaluating the reaction yield; cost of all chemicals employed in the reaction and their safety; the reaction conditions in terms of temperature, time and heating source; and also evaluates the work-up and purification steps.[72]

This work

The penalty points for each parameter were given according to the directives described by Van Aken *et al*.[72] The price of the chemicals employed described in the Eco-scale calculation were extracted from Sigma-Aldrich.[77]

Table S2. Penalty points calculation according to Eco-scale parameter for the MW-assisted esterification of levulinic acid with hexanol**.**

Calculation of penalty points		
	**Parameter**	**Penalty points**
**1**	**Reaction yield** (Yield = 98.85 %)	0.575
**2**	**Price of reaction components (to obtain 10 mmol hexyl levulinate)**<br>Levulinic acid: 0.20 $; hexanol: 0.55 $; *p*-toluenesulfonic acid: 0.02 $; $NaHCO_3$: 0.09 $	0
**3**	**Safety.** No safety statements related to the chemicals employed	0
**4**	**Technical setup.** Unconventional activation technique (microwave irradiation)	2
**5**	**Temperature/time.** Heating, < 1 h (200 °C, 5 min)	2
**6**	**Workup and purification**	
	Removal of solvent with bp > 150°C (hexanol, bp 157 °C)	2
	Distillation	3
	Liquid-liquid extraction/washing ($NaHCO_3$ washing)	3
**Total penalty points**		**12.575**

4. Side reactions assessment

Levulinic acid auto aldol condensation

In acidic media, levulinic acid can undergo autocondensation via aldol condensation following different pathways, as is depicted in **Scheme S2**.[55, 56] The main pathway is that which result in the formation of product 4, as was described by Amaransekara *et al*.[55]

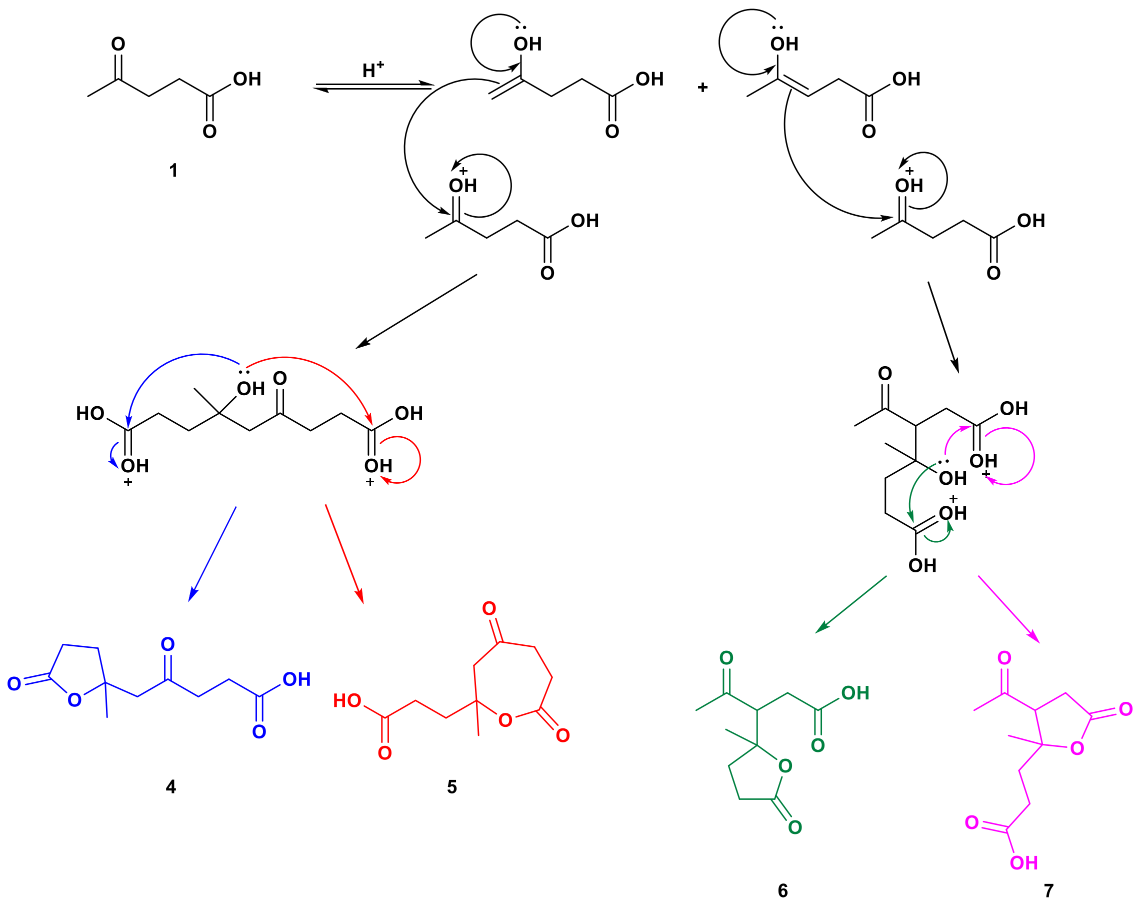

Scheme S2. Possible reaction pathways for levulinic acid aldol condensation according to Amaransekara *et al*. [55].

The formation of product **4** was observed (**Figure S3**) performing the reaction in absence of alcohol, only with levulinic acid and *p*-TSA (5 wt.%) under the optimized conditions (microwave radiation at 200 °C for 5 minutes). The singlets at 2.84 ppm and 1.40 ppm correspond to the presence of product **4**. In addition, other minor signals can be observed

around the singlet corresponding to levulinic acid methyl group (2.13 ppm) and the aforementioned signal at 1.40 ppm, which could be due to the formation of the other products depicted in **Scheme S2**.

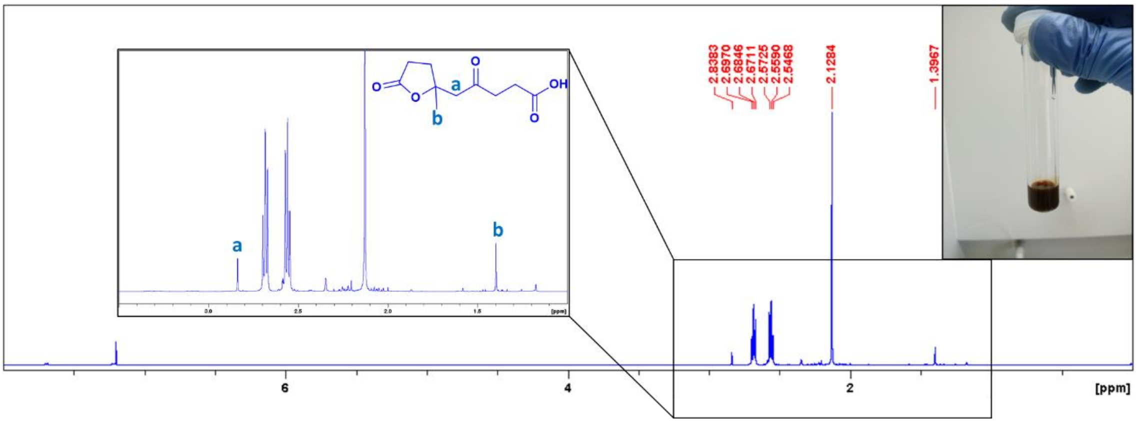

Figure S3. ^{1}H NMR spectrum (500 MHz, $CDCl_3$) of reaction in absence of alcohol (levulinic acid + *p*-TSA 5 wt.%).

That spectrum was compared with that for all reactions carried out, and in **Figures S4-S5** are shown the ^{1}H NMR spectra of two crude reaction in which the aldol condensation of levulinic acid could be more favourable: high levulinic acid concentration (molar ratio LA:alcohol 1:2) (**Figure S4**) and long reaction times (molar ratio LA:alcohol 1:6 and 90 minutes) (**Figure S5**). It can be observed that the signals of product 4 are not present in these spectra, and therefore it could be inferred that in this reaction system, levulinic acid only undergoes esterification with the corresponding alcohol.

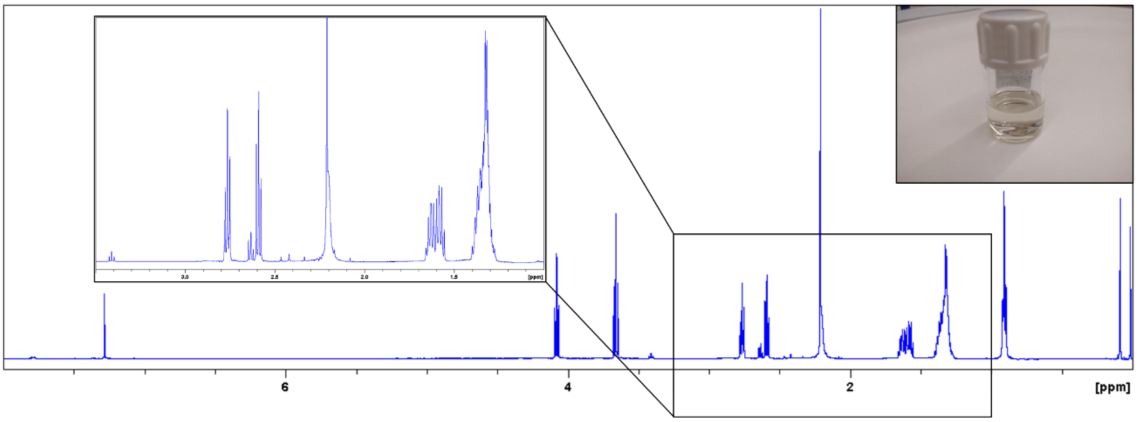

Figure S4. ^{1}H NMR spectrum (500 MHz, $CDCl_3$) of levulinic acid esterification with hexanol (molar ratio LA:alcohol 1:2).

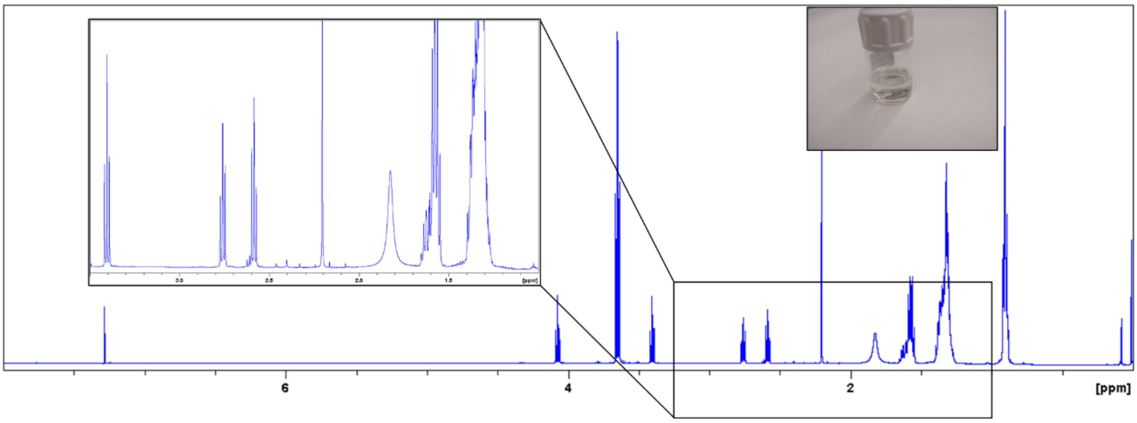

Figure S5. ^{1}H NMR spectrum (500 MHz, $CDCl_3$) of levulinic acid esterification with hexanol (molar ratio LA:alcohol 1:6 and 90 minutes).

Acetal/hemiacetal formation

Another reaction that levulinic acid can undergo in acidic media in presence of an alcohol is the formation of acetal or hemiacetal in the ketone (**Figure 1d**). To assess the possible formation acetals under the reaction conditions studied in this work, 2-butanone was subjected to MW-assisted treatment with hexanol for two different times: 5 minutes (optimal conditions), and 90 minutes (longest reaction time studied in this work). As can be seen in **Figure S6**, the appearance of new signals of 2-butanone due to the formation of the corresponding

acetal/hemiacetal was not observed. The only reaction product observed after 90 minutes was dihexyl ether (triplet at 3.4 ppm).

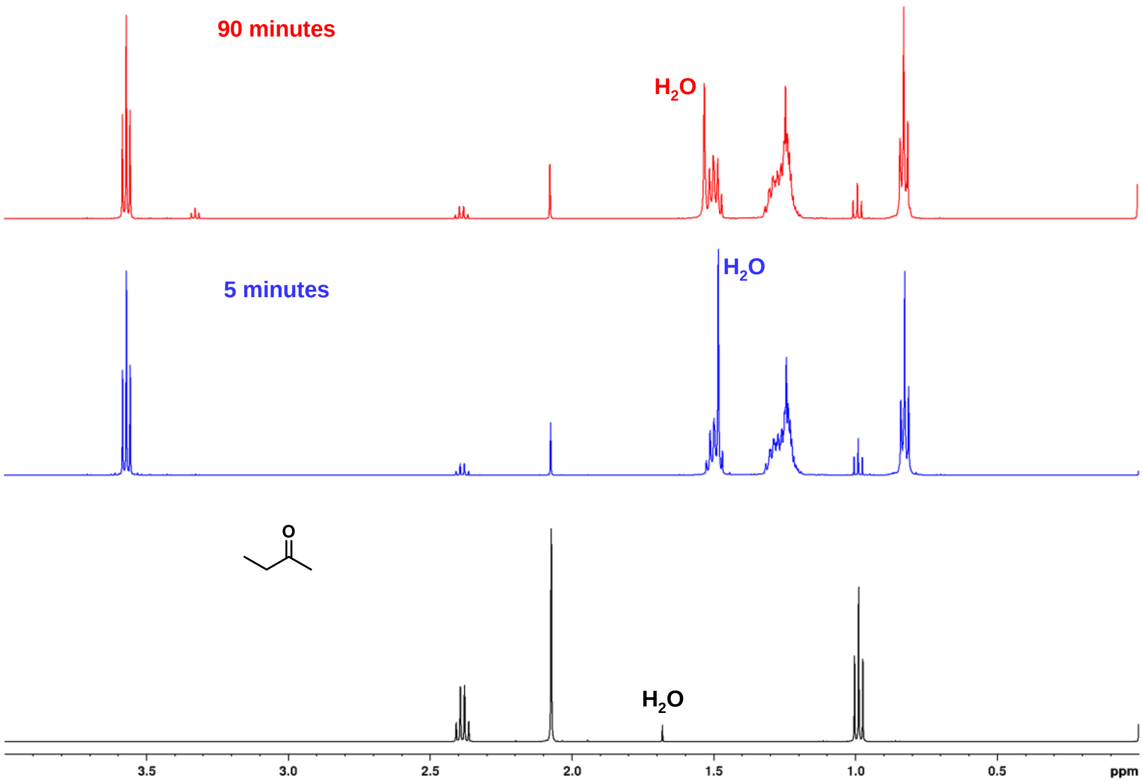

Figure S6. Stacked ^{1}H NMR spectra (500 MHz, $CDCl_3$) of 2-butanone (black), 2-butanone + hexanol 5 minutes reaction time (blue), and 2-butanone + hexanol 90 minutes reaction time (red). Molar ratio 2-butanone:hexanol 1:6, 200 °C, *p*-TSA 5 wt.%.

5. Hexanol recovery

The difference in boiling point between hexanol (157 °C) and the other reaction components, namely hexyl levulinate (277 °C), levulinic acid (245 °C) and dihexyl ether (229 °C), allowed the recovery of unreacted hexanol by means of simple distillation. Two fractions were collected, the first at 100 °C which correspond to the water formed in the reaction. The second fraction was collected at 157 °C, which correspond to unreacted hexanol as a viscous and colorless liquid (**Figure S7).** Considering the hexyl levulinate yield and hexanol conversion to dihexyl ether, it was possible to know the amount of hexanol which has reacted and then, subtracting the hexanol which reacted to the total amount of hexanol, know the theoretical amount of unreacted hexanol. The theoretical unreacted hexanol after carrying out the reaction calculated was 6.52 g, obtaining by distillation 5.74 g of optimal purity hexanol (**Figure S8).** This corresponds to a recovery of 88 % over the total amount of unreacted hexanol.

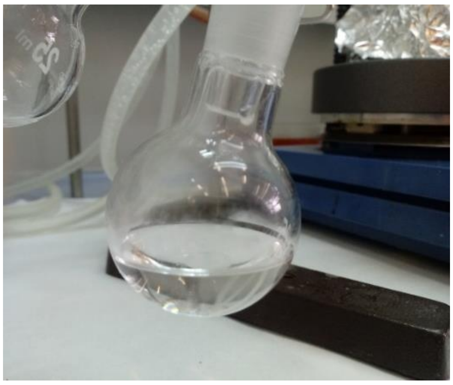

Figure S7. Hexanol recovered by distillation.

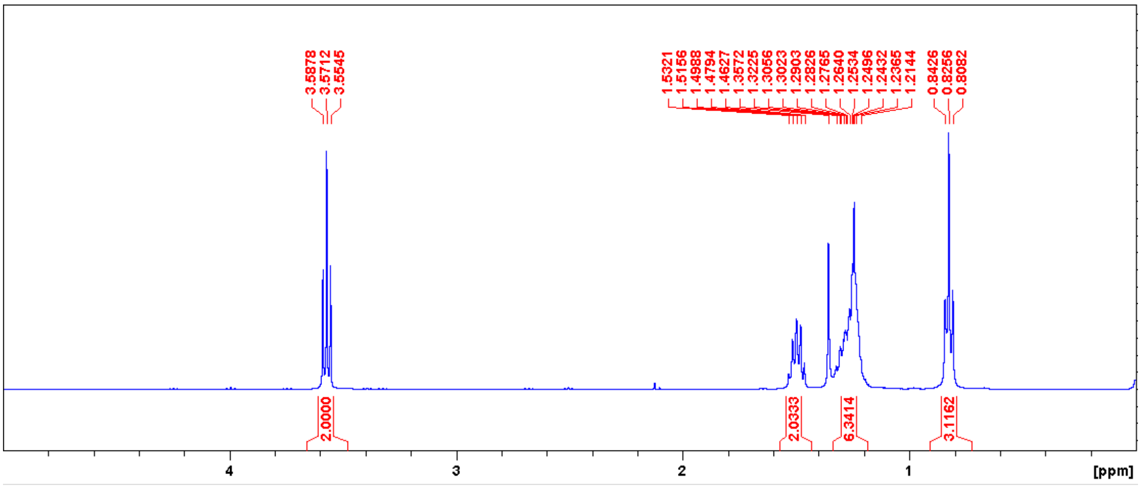

Figure S8. ^{1}H NMR spectrum (500 MHz, $CDCl_3$) of recovered hexanol.

6. ^{1}H NMR and ^{13}C NMR spectra of products

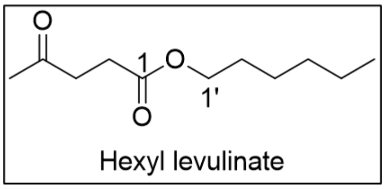

Hexyl levulinate (3a) **^{1}H NMR** (500 MHz, $CDCl_3$) δ (ppm) 4.06 (1'-CH_2, t, 2H), 2.75 (3-CH_2, t, 2H), 2.58 (2-CH_2, t, 2H), 2.19 (5-CH_3, s, 3H), 1.64-1.58 (2'-CH_2, m, 2H), 1.37-1.27 (3'-5'-CH_2, m, 6H), 0.89 (6'-CH_3, t, 3H). **^{13}C{^{1}H} NMR** (126 MHz, $CDCl_3$) δ (ppm) 205.6 (4-C=O), 171.8 (1-COO-), 63.81(1'-CH_2-O-), 36.9, 30.4, 28.8, 27.5, 27.0, 24.5, 21.5, 13.0 (6'-CH_3).

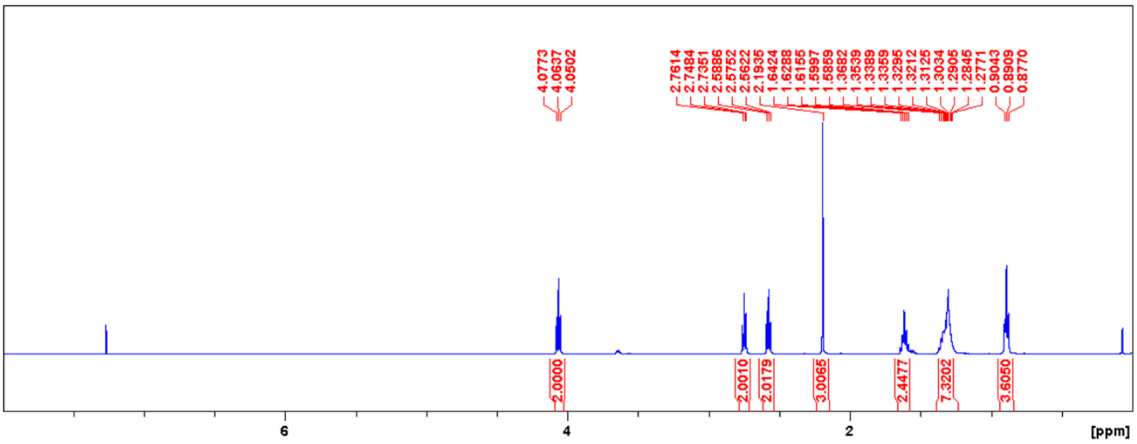

Figure S9. ^{1}H NMR spectrum (500 MHz, $CDCl_3$) of hexyl levulinate.

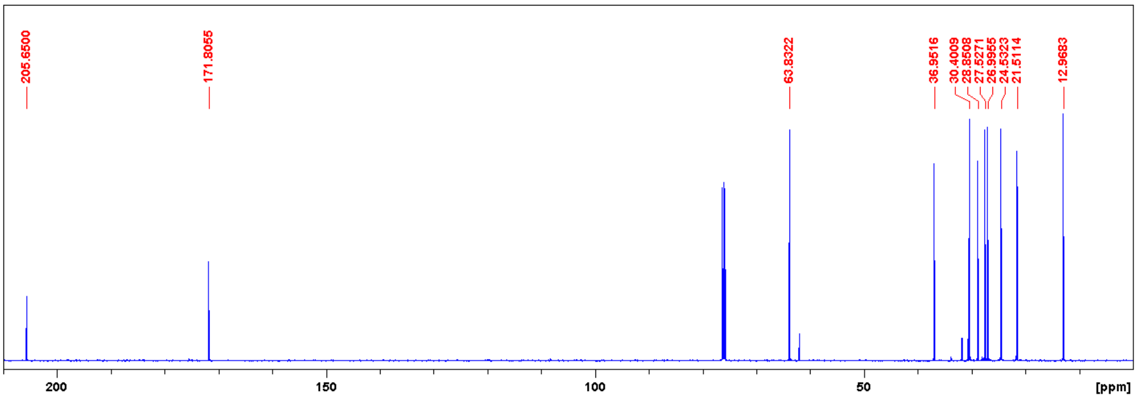

Figure S10. ^{13}C NMR spectrum (126 MHz, $CDCl_3$) of hexyl levulinate.

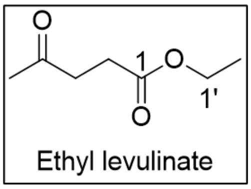

Ethyl levulinate (3b) **^{1}H NMR** (500 MHz, $CDCl_3$) δ (ppm) 4.13 (1'-CH_2, q, 2H), 2.75 (3-CH_2, t, 2H), 2.57 (2-CH_2, t, 2H), 2.19 (5-CH_3, s, 3H), 1.25 (2'-CH_3, t, 3H). **$^{13}C\{^1H\}$ NMR** (126 MHz, $CDCl_3$) δ (ppm) 205.7 (4-C=O), 171.7 (1-COO-), 59.6 (1'-CH_2-O-), 36.9, 28.8, 27.0, 13.1 (2'-CH_3).

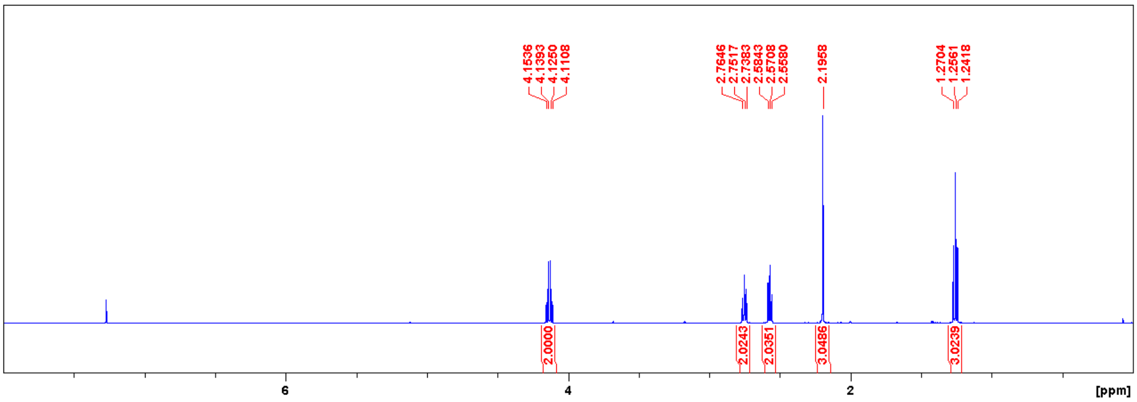

Figure S11. ^{1}H NMR spectrum (500 MHz, $CDCl_3$) of ethyl levulinate.

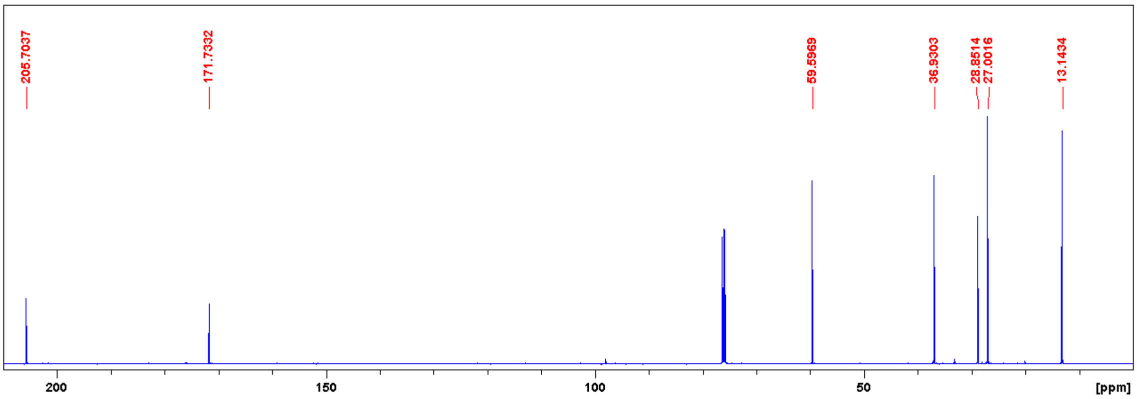

Figure S12. ^{13}C NMR spectrum (126 MHz, $CDCl_3$) of ethyl levulinate.

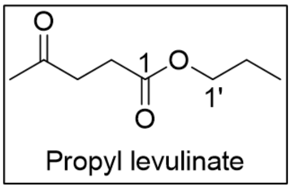

Propyl levulinate (3c) **^{1}H NMR** (500 MHz, $CDCl_3$) δ (ppm) 4.03 (1'-CH_2, t, 2H), 2.75 (3-CH_2, t, 2H), 2.58 (2-CH_2, t, 2H), 2.19 (5-CH_3, s, 3H), 1.68-1.61 (2'-CH_2, m, 2H), 0.94 (3'-CH_3, t, 3H). **$^{13}C\{^1H\}$ NMR** (126 MHz, $CDCl_3$) δ (ppm) 205.7 (4-C=O), 171.8 (1-COO-), 65.2 (1'-CH_2-O-), 36.9, 28.8, 27.0, 20.9, 9.3 (3'-CH_3).

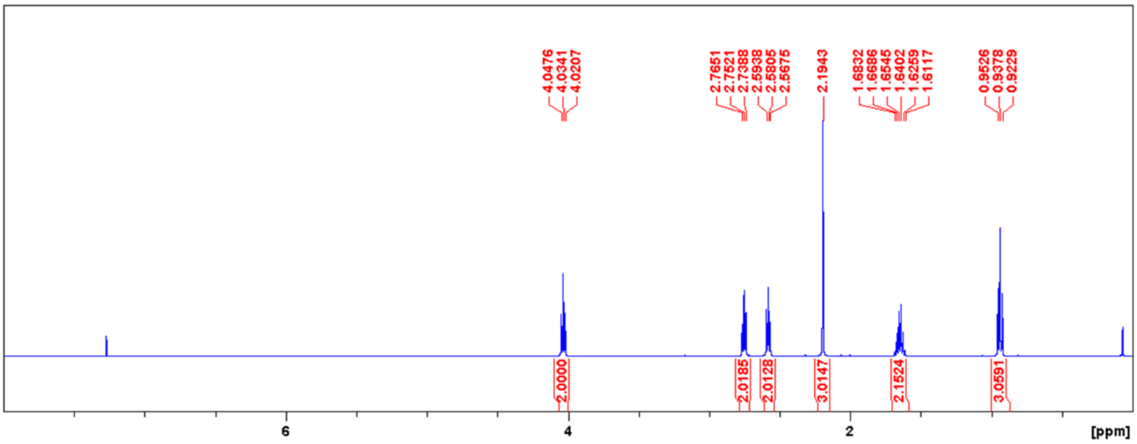

Figure S13. ^{1}H NMR spectrum (500 MHz, $CDCl_3$) of propyl levulinate.

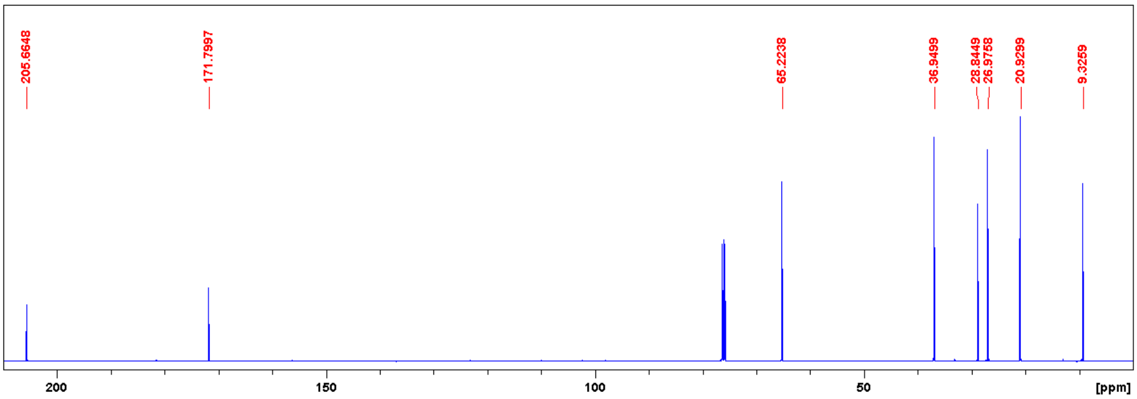

Figure S14. ^{13}C NMR spectrum (126 MHz, $CDCl_3$) of propyl levulinate.

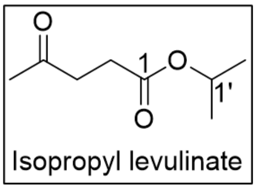

Isopropyl levulinate (3d) **^{1}H NMR** (500 MHz, $CDCl_3$) δ (ppm) 5.03-4.95 (1'-CH_2, m, 1H), 2.74 (3-CH_2, t, 2H), 2.54 (2-CH_2, t, 2H), 2.19 (5-CH_3, s, 3H), 1.23 (2'-CH_3, d, 6H). **^{13}C{^{1}H} NMR** (126 MHz, $CDCl_3$) δ (ppm) 206.7 (4-C=O), 172.2 (1-COO-), 67.9 (1'-CH-O), 38.0, 29.8, 28.3, 21.7 (2'-CH_3, 2C).

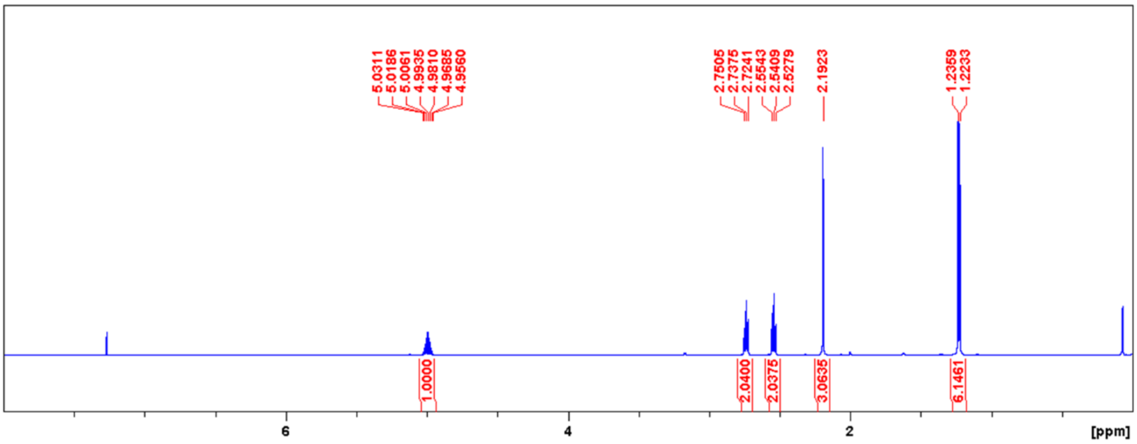

Figure S15. ^{1}H NMR spectrum (500 MHz, $CDCl_3$) of isopropyl levulinate.

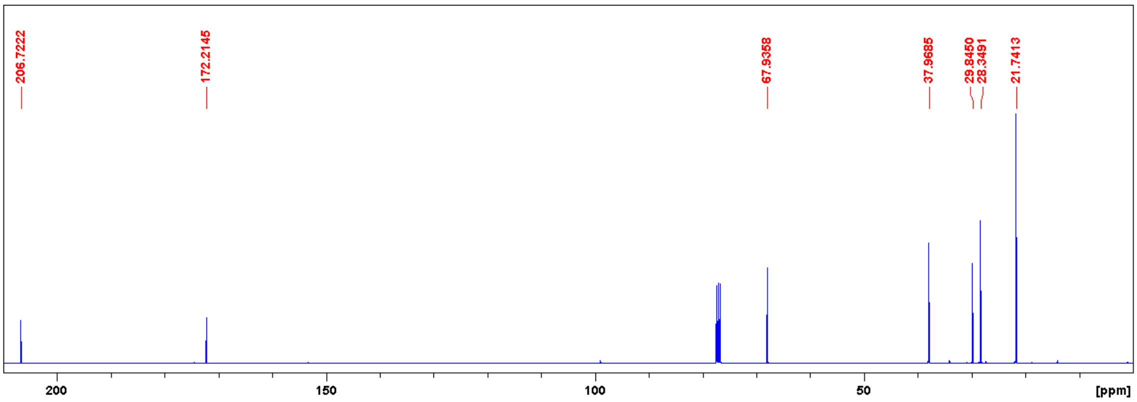

Figure S16. ^{13}C NMR spectrum (126 MHz, $CDCl_3$) of isopropyl levulinate.

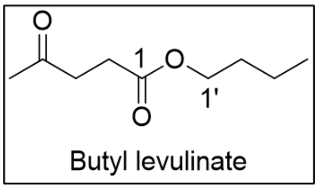

Butyl levulinate (3e) ^{1}**H NMR** (500 MHz, $CDCl_3$) δ (ppm) 4.08 (1'-CH_2, t, 2H), 2.75 (3-CH_2, t, 2H), 2.57 (2-CH_2, t, 2H), 2.19 (5-CH_3, s, 3H), 1.63-1.57 (2'-CH_2, m, 2H), 1.41-1.33 (3'-CH_2, m, 2H), 0.93 (4'-CH_3, t, 3H). ^{13}**C{**^{1}**H} NMR** (126 MHz, $CDCl_3$) δ (ppm) 205.7 (4-C=O), 171.8 (1-COO-), 63.5 (1'-CH_2-O), 36.9, 29.6, 28.8, 27.0, 18.1, 12.7 (4'-CH_3).

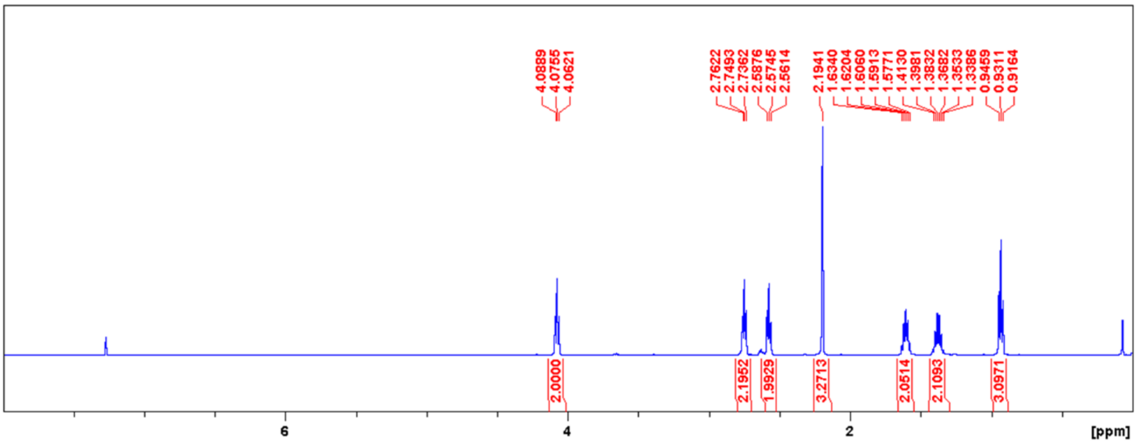

Figure S17. ^{1}H NMR spectrum (500 MHz, $CDCl_3$) of butyl levulinate.

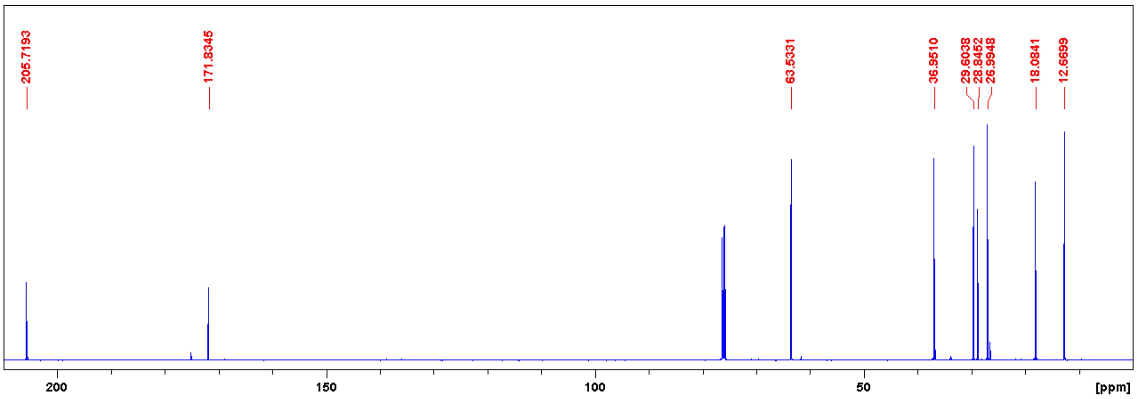

Figure S18. ^{13}C NMR spectrum (126 MHz, $CDCl_3$) of butyl levulinate.

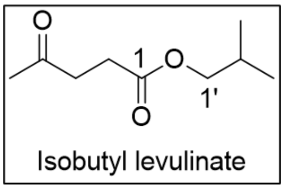

Isobutyl levulinate (3f) **^{1}H NMR** (500 MHz, $CDCl_3$) δ (ppm) 3.86 (1'-CH_2, d, 2H), 2.76 (3-CH_2, t, 2H), 2.59 (2-CH_2, t, 2H), 2.19 (5-CH_3, s, 3H), 1.99-1.85 (2'-CH_2, m, 1H), 0.92 (3'-CH_3, d, 6H). **^{13}C{^{1}H} NMR** (126 MHz, $CDCl_3$) δ (ppm) 205.7 (4-C=O), 171.8 (1-COO-), 69.7 (1'-CH_2-O), 37.0, 28.8, 27.0, 26.7, 18.0 (3'-CH_3, 2C).

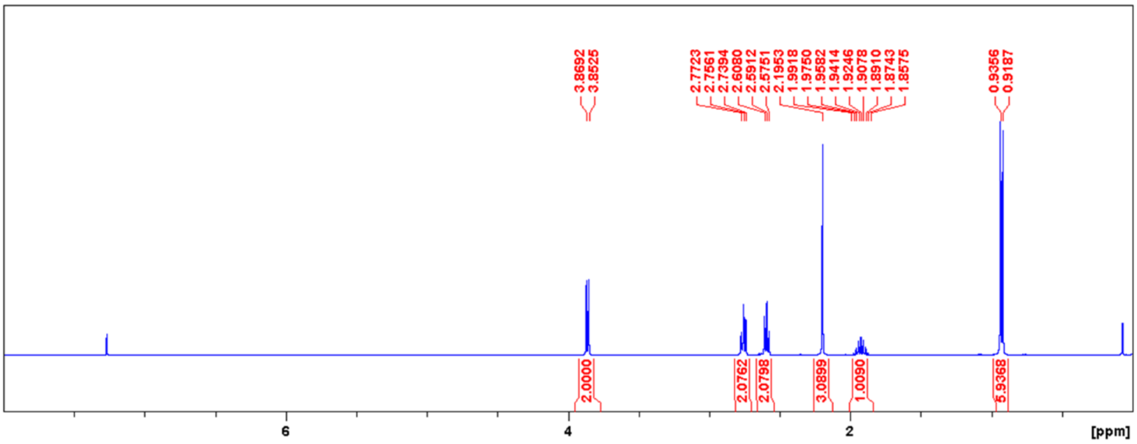

Figure S19. ^{1}H NMR spectrum (500 MHz, $CDCl_3$) of isobutyl levulinate.

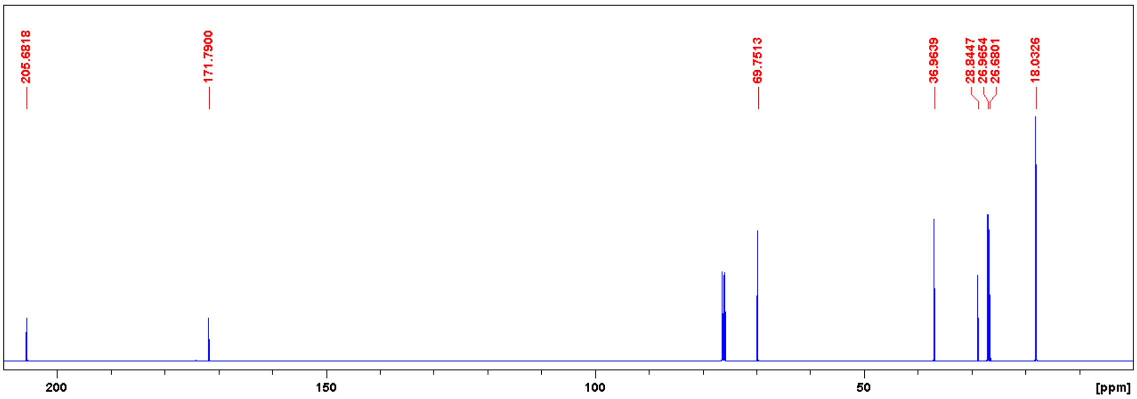

Figure S20. ^{13}C NMR spectrum (126 MHz, $CDCl_3$) of isobutyl levulinate.

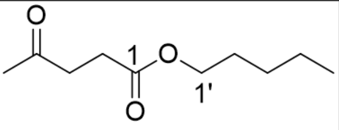

Pentyl levulinate

Pentyl levulinate (3g) **^{1}H NMR** (500 MHz, $CDCl_3$) δ (ppm) 4.06 (1'-CH_2, t, 2H), 2.75 (3-CH_2, t, 2H), 2.57 (2-CH_2, t, 2H), 2.19 (5-CH_3, s, 3H), 1.65-1.58 (2'-CH_2, m, 2H), 1.34-1.31 (3', 4'-CH_2, m, 4H), 0.90 (5'-CH_3, t, 3H). **^{13}C{^{1}H} NMR** (126 MHz, $CDCl_3$) δ (ppm) 206.7 (4-C=O), 172.8 (1-COO-), 64.8 (1'-CH_2-O), 38.0, 29.9, 28.3, 28.0, 28.0, 22.3, 14.0 (5'-CH_3).

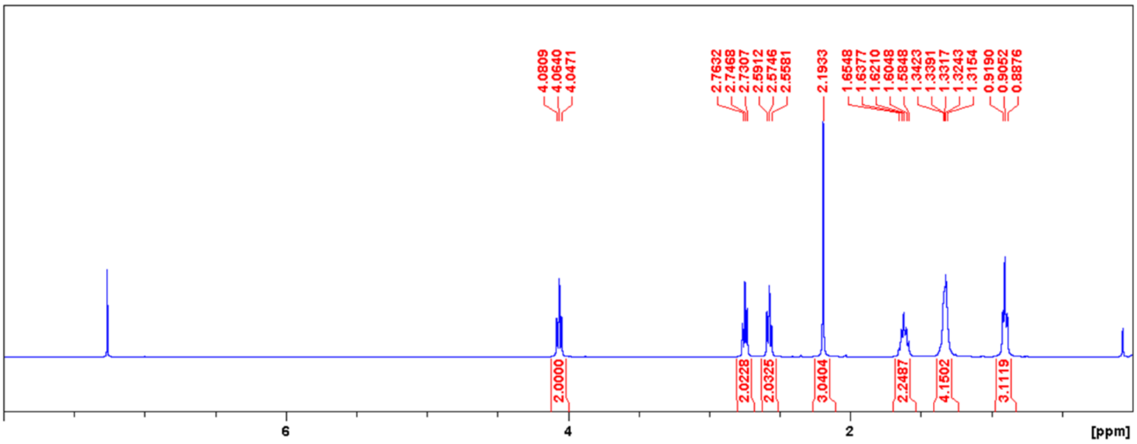

Figure S21. ^{1}H NMR spectrum (500 MHz, $CDCl_3$) of pentyl levulinate.

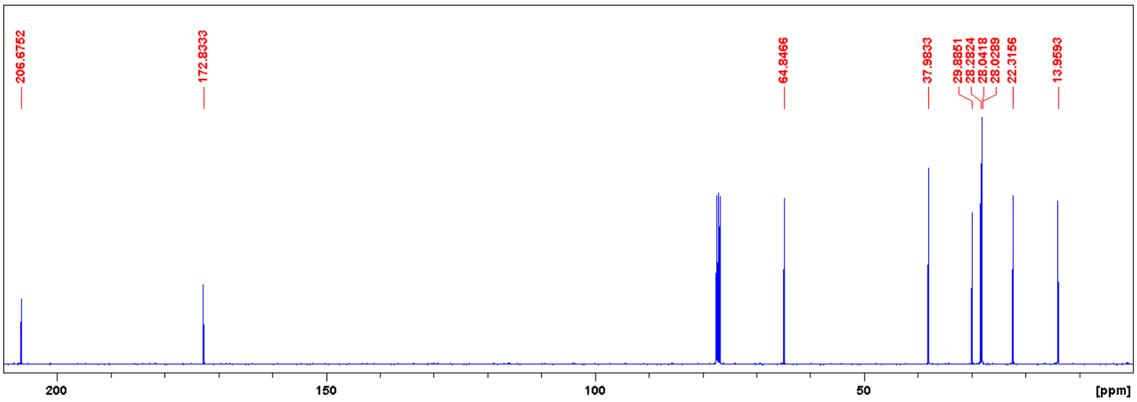

Figure S22. ^{13}C NMR spectrum (126 MHz, $CDCl_3$) of pentyl levulinate.

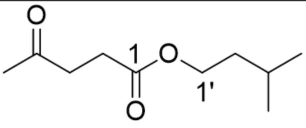

Isopentyl levulinate

Isopentyl levulinate (3h) **^{1}H NMR** (500 MHz, $CDCl_3$) δ (ppm) 4.10 (1'-CH_2, t, 2H), 2.75 (3-CH_2, t, 2H), 2.57 (2-CH_2, t, 2H), 2.19 (5-CH_3, s, 3H), 1.72-1.64 (3'-CH_2, m, 1H), 1.54-1.49 (2'-CH_2, m, 2H), 0.92 (4'-CH_3, d, 6H). **^{13}C{^{1}H} NMR** (126 MHz, $CDCl_3$) δ (ppm) 206.7 (4-C=O), 172.8 (1-COO-), 63.3 (1'-CH_2-O), 37.9, 37.2, 29.8, 28.0, 25.0, 22.4 (4'-CH_3, 2C).

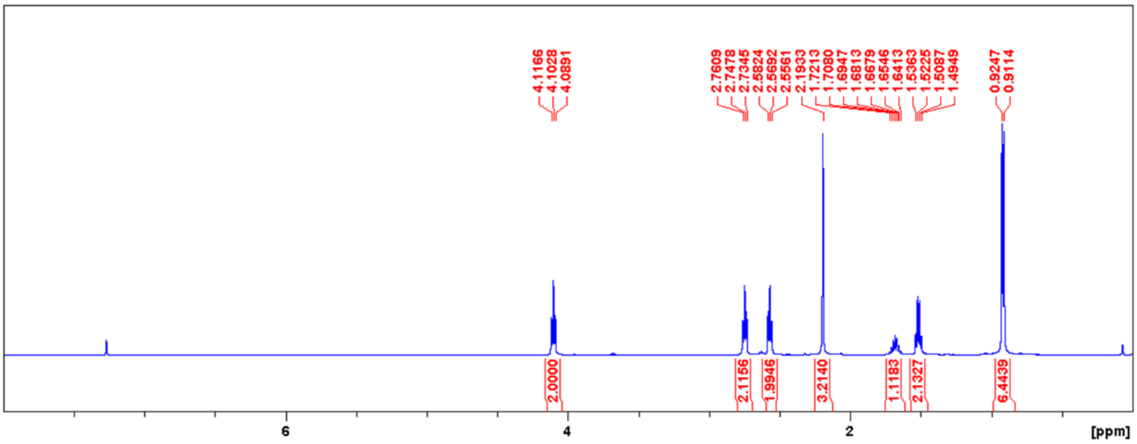

Figure S23. ^{1}H NMR spectrum (500 MHz, $CDCl_3$) of isopentyl levulinate.

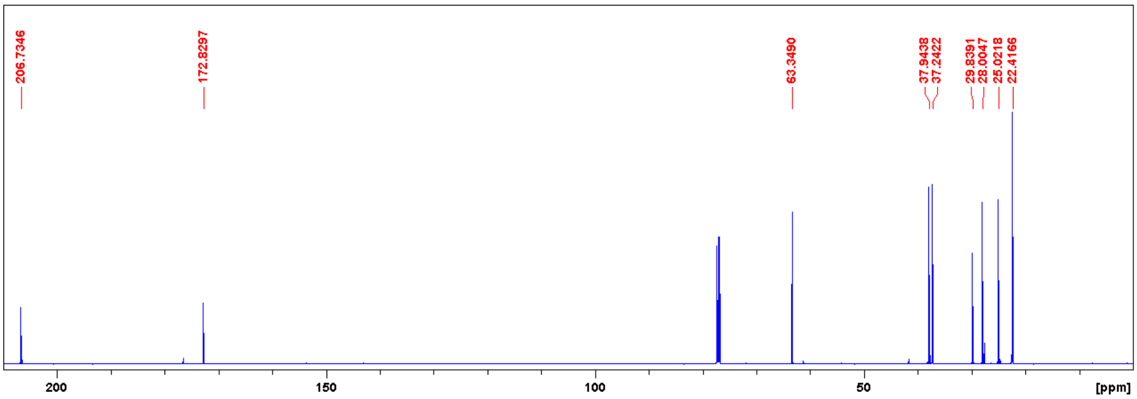

Figure S24. ^{13}C NMR spectrum (126 MHz, $CDCl_3$) of isopentyl levulinate.

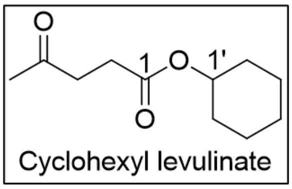

Cyclohexyl levulinate (3i) **^{1}H NMR** (500 MHz, $CDCl_3$) δ (ppm) 4.77-4.71 (1'-CH, m, 1H), 2.74 (3-CH_2, t, 2H), 2.56 (2-CH_2, t, 2H), 2.19 (5-CH_3, s, 3H), 1.84-1.23 (2'-4'-CH_2, m, 10H). **^{13}C{^{1}H} NMR** (126 MHz, $CDCl_3$) δ (ppm) 205.7 (4-C=O), 171.1 (1-COO-), 71.9 (1'-CH-O), 37.0, 30.5, 28.9, 27.4, 24.3, 22.7.

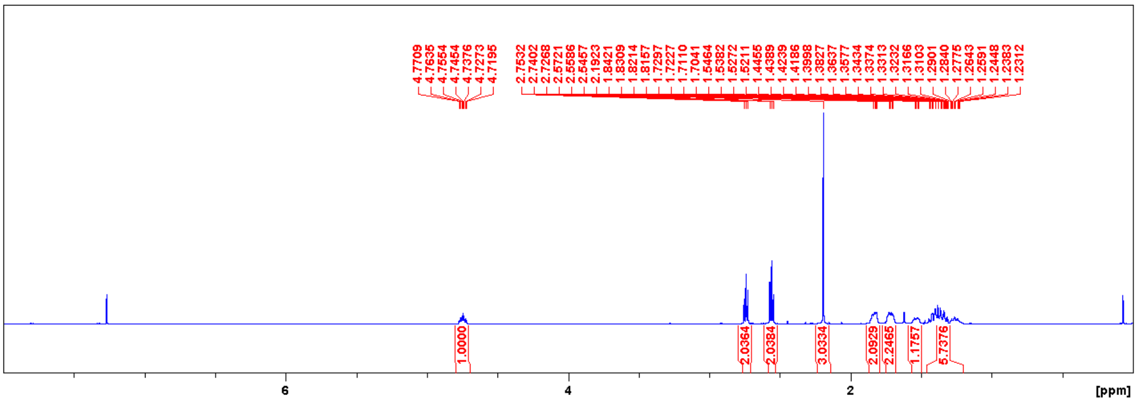

Figure S25. ^{1}H NMR spectrum (500 MHz, $CDCl_3$) of cyclohexyl levulinate.

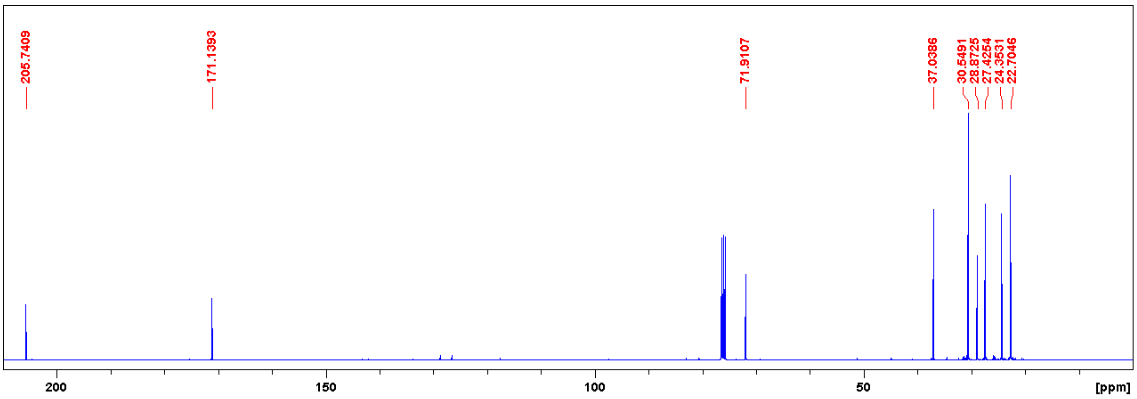

Figure S26. ^{13}C NMR spectrum (126 MHz, $CDCl_3$) of cyclohexyl levulinate.

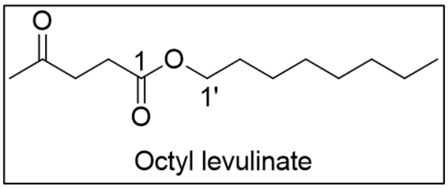

Octyl levulinate (3j) **^{1}H NMR** (500 MHz, $CDCl_3$) δ (ppm) 4.06 (1'-CH_2, t, 2H), 2.75 (3-CH_2, t, 2H), 2.57 (2-CH_2, t, 2H), 2.19 (5-CH_3, s, 3H), 1.64-1.58 (2'-CH_2, m, 2H), 1.34-1.27 (3'-7'-CH_2, m, 10H), 0.88 (8'-CH_3, t, 3H). **^{13}C{^{1}H} NMR** (126 MHz, $CDCl_3$) δ (ppm) 206.6 (4-C=O), 172.8 (1-COO-), 64.8 (1'-CH_2-O), 37.9, 31.7, 29.8, 29.2, 29.1, 28.6, 28.0, 25.9, 22.6, 14.1 (8'-CH_3).

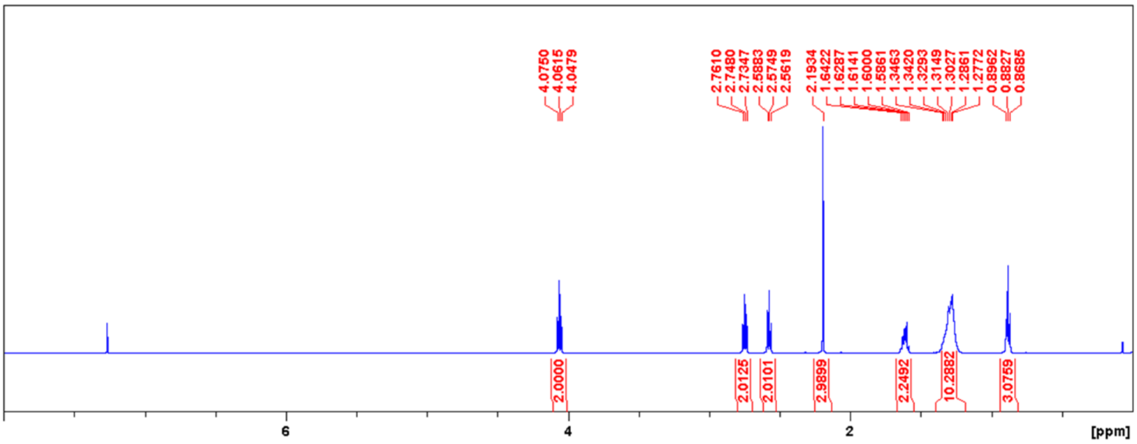

Figure S27. ^{1}H NMR spectrum (500 MHz, $CDCl_3$) of octyl levulinate.

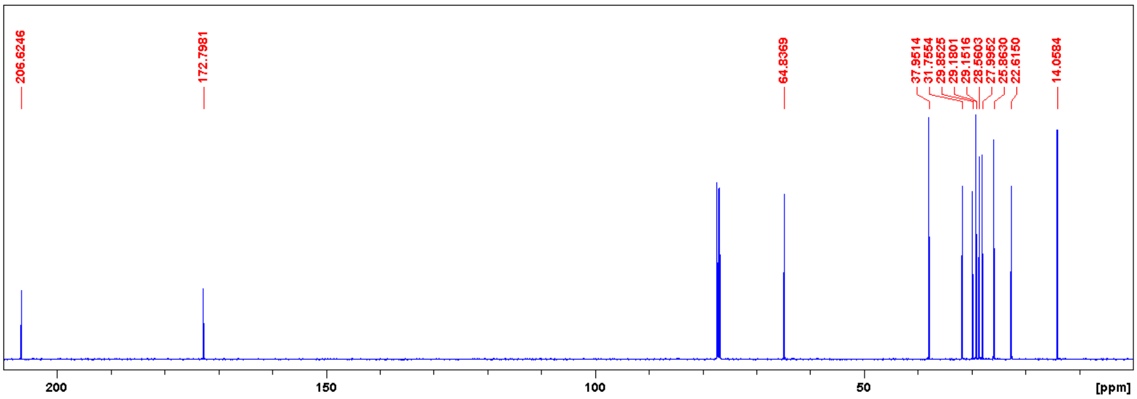

Figure S28. ^{13}C NMR spectrum (126 MHz, $CDCl_3$) of octyl levulinate.

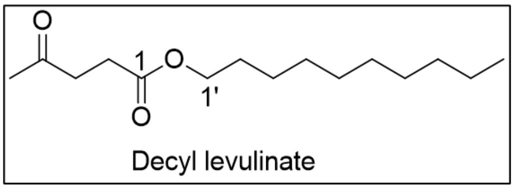

Decyl levulinate (3k) ^{1}H NMR (500 MHz, $CDCl_3$) δ (ppm) 4.06 (1'-CH_2, t, 2H), 2.75 (3-CH_2, t, 2H), 2.57 (2-CH_2, t, 2H), 2.19 (5-CH_3, s, 3H), 1.64-1.59 (2'-CH_2, m, 2H), 1.34-1.26 (3'-7'-CH_2, m, 14H), 0.88 (8'-CH_3, t, 3H). **^{13}C{^{1}H} NMR** (126 MHz, $CDCl_3$) δ (ppm) 205.6 (4-C=O), 171.8 (1-COO-), 63.8 (1'-CH_2-O), 36.9, 30.9, 28.8, 28.5, 28.5, 28.3, 28.2, 27.8, 27.0, 24.9, 21.7, 13.1 (10'-CH_3).

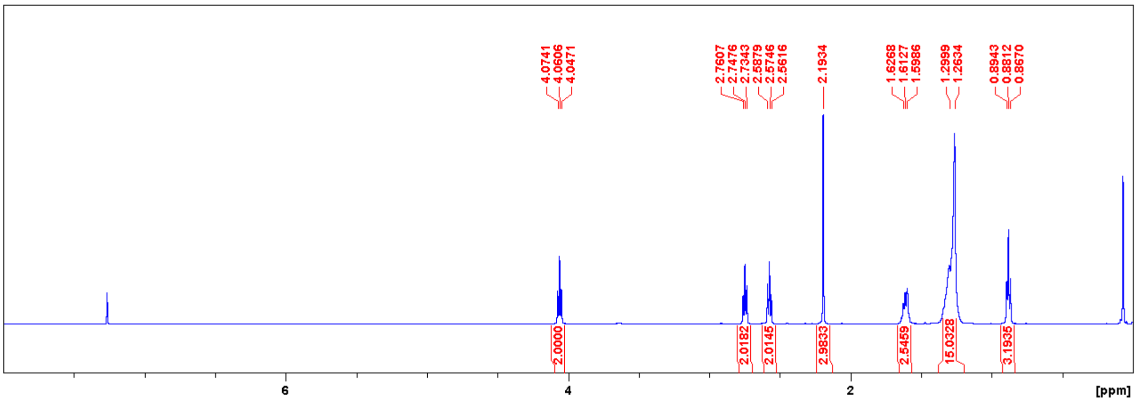

Figure S29. ^{1}H NMR spectrum (500 MHz, $CDCl_3$) of decyl levulinate.

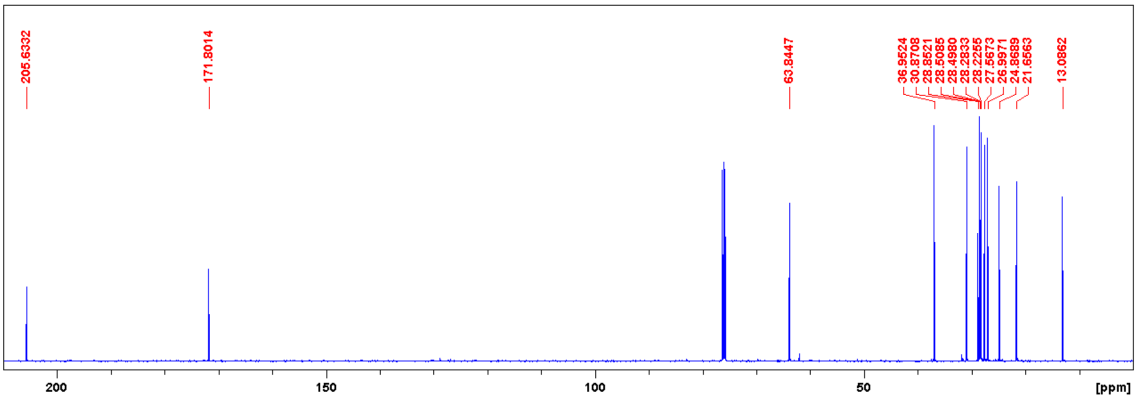

Figure S30. ^{13}C NMR spectrum (126 MHz, $CDCl_3$) of decyl levulinate.

7. References

[55] A. S. Amarasekara, B. Wiredu, T. L. Grady, R. G. Obregon, D. Margetić, *Catal. Commun.* **2019**, *124*, 6–11.

[56] M. Paniagua, F. Cuevas, G. Morales, J. A. Melero, *ACS Sustainable Chem. Eng.* **2021**, *9*, 5952–5962.

[72] K. V. Aken, L. Strekowski, L. Patiny, *Beilstein J. Org. Chem*. **2006**, *2*, 3.

[73] C. Cativiela, J. M. Fraile, J. I. García, J. A. Mayoral, E. Pires, A. J. Royo, F. Figueras, L. C. de Ménorval, *Tetrahedron* **1993**, *49*, 4073–4084.

[74] U. Holzgrabe, *Prog. Nucl. Magn. Reson. Spectrosc*. **2010**, *57*, 229–240.

[75] M. Salgado-Ramos, S. Tabasso, E. C. Gaudino, G. Cravotto, *Ind. Eng. Chem. Res*. **2024**, *63*, 7507–7518.

[76] N. Fantozzi, J.-N. Volle, A. Porcheddu, D. Virieux, F. García, E. Colacino, *Chem. Soc. Rev*. **2023**, *52*, 6680–6714.

[77] MilliporeSigma. Life Science Products & Service Solutions, can be found under https://www.sigmaaldrich.com/US/en, **2024**.
